# Neutrophil-fibroblast crosstalk drives immunofibrosis in Crohn’s disease through IFNα pathway

**DOI:** 10.3389/fimmu.2024.1447608

**Published:** 2024-09-13

**Authors:** Efstratios Gavriilidis, Georgios Divolis, Anastasia-Maria Natsi, Nikolaos Kafalis, Dionysios Kogias, Christina Antoniadou, Evgenia Synolaki, Evgenios Pavlos, Marianna A. Koutsi, Stylianos Didaskalou, Evangelos Papadimitriou, Victoria Tsironidou, Ariana Gavriil, Vasileios Papadopoulos, Marios Agelopoulos, Dimitrios Tsilingiris, Maria Koffa, Alexandra Giatromanolaki, Georgios Kouklakis, Konstantinos Ritis, Panagiotis Skendros

**Affiliations:** ^1^ First Department of Internal Medicine, University Hospital of Alexandroupolis, Democritus University of Thrace, Alexandroupolis, Greece; ^2^ Laboratory of Molecular Hematology, Department of Medicine, Democritus University of Thrace, Alexandroupolis, Greece; ^3^ Center for Clinical, Experimental Surgery and Translational Research, Biomedical Research Foundation Academy of Athens, Athens, Greece; ^4^ Gastroenterology-Hepatology Unit, University Hospital of Alexandroupolis, Alexandroupolis, Greece; ^5^ Center of Basic Research, Biomedical Research Foundation Academy of Athens, Athens, Greece; ^6^ Laboratory of Cell Biology, Proteomics and Cell Cycle, Department of Molecular Biology and Genetics, Democritus University of Thrace, Alexandroupolis, Greece; ^7^ Department of Pathology, University Hospital of Alexandroupolis, Democritus University of Thrace, Alexandroupolis, Greece

**Keywords:** immunofibrosis, Crohn’s disease, IFNα, neutrophils, NETs, fibroblasts

## Abstract

**Introduction:**

Crohn’s disease (CD) is characterized by chronic inflammation and intestinal fibrosis leading to lifelong complications. However, the disease pathogenesis remains elusive, and the therapeutic options are limited. Here, we investigated the interaction between neutrophils and intestinal fibroblasts in the development of CD immunofibrosis, a disease mechanism predisposing to inflammatory and fibrotic complications.

**Methods:**

Peripheral neutrophils, enriched neutrophil extracellular traps (eNETs), serum, primary intestinal fibroblasts (PIFs) and intestinal biopsies from CD, ulcerative colitis (UC) patients, and healthy individuals (HI), were studied. Transcriptome analysis of neutrophils, multi-cytokine profiling and cell-based functional assays at mRNA/protein level were performed.

**Results:**

Compared to UC, PIFs from CD patients, independently to the presence of strictures, displayed a distinct pro-fibrotic phenotype characterized by negative Krüppellike Factor-2 (KLF2) and increased cellular communication network factor-2 (CCN2) expression leading to collagen production. In both UC and CD, PIFs-derived IL-8 acted as a culprit chemoattractant for neutrophils in the intestine, where CD neutrophils were accumulated close to fibrotic lesions. Functionally, only CD neutrophils via eNETs induced a CD-like phenotype in HI PIFs, suggesting their fibrotic plasticity. High IFNa in serum and IFΝ-responsive signature in peripheral neutrophils were observed in CD, distinguishing it from UC. Moreover, CD serum stimulated the release of fibrogenic eNETs from neutrophils in an IFNa-dependent manner, suggesting the priming role of IFNa in circulating neutrophils. Inhibition of eNETs or JAK signaling in neutrophils or PIFs prevented the neutrophil-mediated fibrotic effect on PIFs. Furthermore, both serum IFNa levels and mRNA levels of key IFN signaling components in neutrophils were wellcorrelated with CD severity.

**Conclusions:**

This study reveals the important role of the IFNa/neutrophil/fibroblast axis in CD immunofibrosis, suggesting candidate biomarkers and putative therapeutic targets.

## 1 Introduction

Crohn’s disease (CD) is characterized by chronic relapsing inflammation that may lead to intestinal fibrosis causing lifelong disabling illness with a significant impact on the quality of life and the healthcare systems (1, 2). More than 50% of patients develop intestinal strictures in their lifetimes, as a result of ongoing chronic activation of myofibroblasts by the gut inflammatory environment (2). Therefore, understanding the mechanisms of initiation and propagation of intestinal fibrosis in CD is crucial to providing knowledge for diagnosis and better care of patients (3, 4).

Cellular plasticity is fundamental to human immunity and, as recently recognized, a key aspect of neutrophil biology (5, 6). Recent studies suggest that circulating neutrophils, as an adaptation to the different environmental conditions, undergo transcriptional reprogramming that allows them to acquire disease-specific phenotypes and commit their cell-fate plasticity upon their entrance to the site of tissue inflammation attracted by tissue-derived chemotactic factors (6). Activated neutrophils release a plethora of antimicrobial and proinflammatory mediators on extracellular vesicles and traps which dictate their diverse functional role in different diseases (7–9).

In line with these, previous studies indicated that activated neutrophils through the release of neutrophil extracellular traps (NETs) may play a pro-inflammatory or fibrotic role by promoting the differentiation and activation of human fibroblasts (10–12). Recently, our group suggested that NETs and downregulation of transcriptional factor Krüppel-like Factor 2 (KLF2) in human lung fibroblasts are linked with the inflammatory environment of COVID-19 leading to immunofibrosis (13). Whether neutrophils exert immunofibrotic effects on intestinal fibroblasts and how these cells interact with each other in the inflammatory environment of CD leading to tissue damage is largely unknown yet (4, 14).

This study provides a new understanding of the mechanisms involved in CD immunofibrosis through neutrophils/NETs functional plasticity. We identified that, in contrast to UC, neutrophils of CD are primed by IFNα/JAK signaling to commit an active role on intestinal fibroblasts, thus inducing their distinct fibrotic phenotype. The production of IL-8 by intestinal fibroblasts sustains their mutual interaction with neutrophils. Translating these findings, the levels of key IFN type I pathway components are positively correlated with disease severity in CD patients promising novel diagnostic and therapeutic targets.

## 2 Materials and methods

### 2.1 Patients and sampling

The study was conducted in the First Department of Internal Medicine and the Gastroenterology-Hepatology Unit, at the University Hospital of Alexandroupolis. In total 26 treatment-naïve CD (16 male/10 female; mean age 35.3 ± 15.0 years), 32 UC patients (24 male/8 female; mean age 52.2 ± 19.1 years) and 18 healthy individuals, including 4 who underwent preventive screening colonoscopy (HI; 12 male/6 female; mean age 39.1 ± 12.6 years), were recruited. Healthy controls were sex- and age-matched with CD patients. The older age of UC patients may be primarily attributed to the earlier age at the time of diagnosis in CD compared to UC, as described in the literature (15). The diagnosis of UC and CD was according to standard clinical, endoscopic, radiological, and histological criteria (1, 16). Clinical severity and disease behavior scores such as Mayo disease activity index (Mayo-DAI) (17), Crohn’s disease activity index (CDAI) (18) and Montreal score (19) were evaluated in both UC and CD patients by two independent, expert gastroenterologists.

Neutrophils, serum, primary intestinal fibroblasts (PIFs) and intestinal biopsies were collected. The baseline clinical characteristics of patients and samples used in experimental procedures are detailed in **Supplementary Tables S1**-**S3**.

### 2.2 Isolation of peripheral blood neutrophils and serum

Peripheral heparinized blood and serum were collected as previously described (20). Detailed methods are included in the **Supplementary Materials and Methods**.

### 2.3 Isolation, culture, and characterization of human primary intestinal fibroblasts

Detailed methods are included in the **Supplementary Materials and Methods**.

### 2.4 Generation and collection of enriched-neutrophil extracellular traps

A total of 1.5 × 10^6^ neutrophils isolated from UC, CD patients and HI were resuspended in Roswell Park Memorial Institute (RPMI) medium (21875; Thermo Fisher Scientific; Carlsbad, SA, USA) supplemented with 2% heat-inactivated fetal bovine serum (FBS);10082147; Thermo Fischer Scientific), and cultured at 37°C with 5% CO_2_, for 3.5 hours, based on a standard isolation protocol to generate *ex-vivo* NET structures. Similarly, healthy neutrophils were also incubated *in-vitro* in the presence of 5% serum from UC, CD patients and HI, or phorbol 12-myristate 13-acetate (PMA) (40ng/mL; P8139; Sigma-Aldrich, St Louis, MO, USA), a generic inducer of NET release, and cultured in the aforementioned conditions. After incubation a vigorous agitation for 5 min was performed, to detach NET structures and this medium was collected. We omitted the washing step, which was carried out during the classic method of NETs isolation (21–23) in order to collect more inflammatory mediators than NETs (10, 24, 25). This mixture of enriched NETs is hereafter referred to as “eNETs”. Aliquots of eNETs were stored at -80°C until analyzed. Both *ex-vivo* and *in-vitro* generated eNETs were further used in stimulation studies in PIFs at a final concentration of 20%.

To quantify NETs, MPO/DNA complexes were measured in *ex-vivo* and *in-vitro* NETs isolated from 1.5 x 10^6^ neutrophils as previously described. In brief, NETs were captured with a human anti-MPO antibody (1:500 dilution; HM2164; clone 6G3-mouse IgG1; Hycult Biotech; Uden, Netherlands), and an anti-double-stranded DNA antibody was used for DNA detection (Cell Death Detection ELISA Kit; 11544675001; Merck; Kenilworth, New Jersey, USA). Absorbance was measured at 405 nm (26, 27).

The concentrations and time points used to test neutrophil function were optimized before the experiments. All materials used were endotoxin-free, as determined by a Limulus amebocyte assay (E8029; Sigma-Aldrich).

### 2.5 Stimulation and inhibition studies in cultured cells

PIFs or peripheral blood neutrophils were seeded into 6-well culture plates (≈0.8-1 x 10^6^ cells/well for fibroblasts, ≈1.5 x 10^6^ cells/well for neutrophils; Corning Incorporated) in complete DMEM and RPMI medium respectively.

Neutrophils were stimulated with recombinant IFNα2 (1000 U/mL; H6041; Sigma-Aldrich), in order to prime them to release eNETs. PIFs were stimulated with 5% serum from HI, UC, and CD patients, in complete DMEM. To block IFN signaling, a neutralizing mouse monoclonal antibody against human Interferon Alpha (anti-IFNα; 15 μg/mL; 21100-1; R&D Systems); was used in both neutrophils and PIFs. To inhibit Janus kinase signaling (JAK-1 and JAK-2), PIFs and neutrophils were pre-treated (60 min) with baricitinib (2.5 nM; 16707; Cayman Chemical). To dismantle NET structures, CD *ex-vivo* eNETs were pre-incubated (60 min) with DNase I (1 U/mL; EN0525, Thermo Fisher Scientific). To trigger the expression of KLF2, PIFs were further pre-incubated (60 min) with Tannic acid (20 nM; 403040; Sigma-Aldrich), a polyphenolic compound, acting as a potent inducer of KLF2. The total culture period for neutrophils was 3.5 hours.

### 2.6 RNA isolation, cDNA synthesis and RT-qPCR

Detailed methods are included in the **Supplementary Materials and Methods**.

### 2.7 RNA sequencing and bioinformatics analysis

One μg of total RNA was used for the preparation of cDNA libraries, as previously described (28, 29). Sequencing was performed in a single-end manner at the Genome Center of Biomedical Research Foundation Academy of Athens, using the NovaSeq 6000 SP 100c kit (20028401; Illumina), generating 100 bp long reads.

Raw sequence data were uploaded to the Galaxy web platform (30), and standard tools of the public server “usegalaxy.org” were used for subsequent analysis, as previously described (29). HISAT2 (v2.2.1+galaxy1) was applied for the alignment of trimmed reads to the human GRCh37/hg19 genome assembly from the Genome Consortium, using the default parameters. Assessment of uniform read coverage for exclusion of 5’/3’ bias and evaluation of RNA integrity at the transcript level were performed, as previously reported (29). Moreover, absolute de-convolution of human immune cell types was applied in our datasets, according to the Shiny app, https://giannimonaco.shinyapps.io/ABIS (31), to ensure their enrichment in granulocytes gene expression signature (> 95%). Replicates with signatures enriched in contaminating lymphocytes (> 5%) were excluded from the analysis. The identification of Differentially Expressed Genes (DEGs) between HI and patients with either CD or UC was carried out with the DESeq2 algorithm (v2.11.40.7+galaxy2) (32), using the count tables generated from the htseq-count tool (v0.9.1+galaxy1) as input.

Pathway analysis was performed using the GeneCodis4 web-based tool (33). Cutoff values for statistically significant DEGs were baseMean >30 and adjusted p-value (false discovery rate, FDR) <0.05. Gene set enrichment analysis (GSEA) was performed using the GSEA software (University of California, San Diego & Broad Institute, USA), as previously described (34, 35). Human Molecular Signatures Database (Human MSigDB v2023.1) was used as input.

Heatmaps were generated using the Morpheus software, https://software.broadinstitute.org/morpheus (Broad Institute, USA). Venn diagrams were created with Venny 2.1 (developed by Oliveros, J.C., 2007) and Venn Diagram Plotter software (Pacific Northwest National Laboratory, U.S. Department of Energy).

### 2.8 In-cell ELISA (ICE assay, Cytoblot)

Details are included in the **Supplementary Materials and Methods**.

### 2.9 Immunofluorescence staining in human PIFs

Details are included in the **Supplementary Materials and Methods**.

### 2.10 Collagen measurement

Details are included in the **Supplementary Materials and Methods**.

### 2.11 Multiplex cytokine measurement

Details are included in the **Supplementary Materials and Methods**.

### 2.12 Immunohistochemistry, Masson’s trichrome and Immunofluorescence staining in tissue sections.

Detailed methods are included in the **Supplementary Materials and Methods**.

### 2.13 *In-vitro* transwell migration assay (chemotaxis assay)

Details are included in the **Supplementary Materials and Methods**.

### 2.14 Statistical analysis

Statistical analysis was performed with the GraphPad Prism software (version 9.0, San Diego, CA, USA). For comparisons involving more than two groups, the nonparametric Kruskal-Wallis test, followed by Dunn’s test, was performed. To compare the migratory capacity of HI PIFs supernatants to those of UC and CD, Bayesian unpaired t-test was used; p-values were adjusted using the Benjamini-Hochberg correction. For comparisons between IL-8 neutralized PIFs supernatants and untreated supernatants, Bayesian paired t-tests were performed, using the JZS Bayes factor (36). Data are expressed as the mean ± standard error of the mean (SEM). Simple linear regression was used to assess the relationship between two variables. The levels of significance were set as follows: *p<0.05, **p<0.01, ***p<0.001, ****p<0.0001.

## 3 Results

### 3.1 Fibroblasts of Crohn’s disease patients display a distinct fibrotic phenotype

Since fibrotic complications are a prominent feature of CD (2, 3) and recent data have shown that the profibrotic activity of human lung fibroblasts is associated with downregulated KLF2, elevated CCN2 levels, and increased collagen production (13), we aimed to investigate whether intestinal fibroblasts in CD share similarities with this fibrotic phenotype.

**Figure 1:**
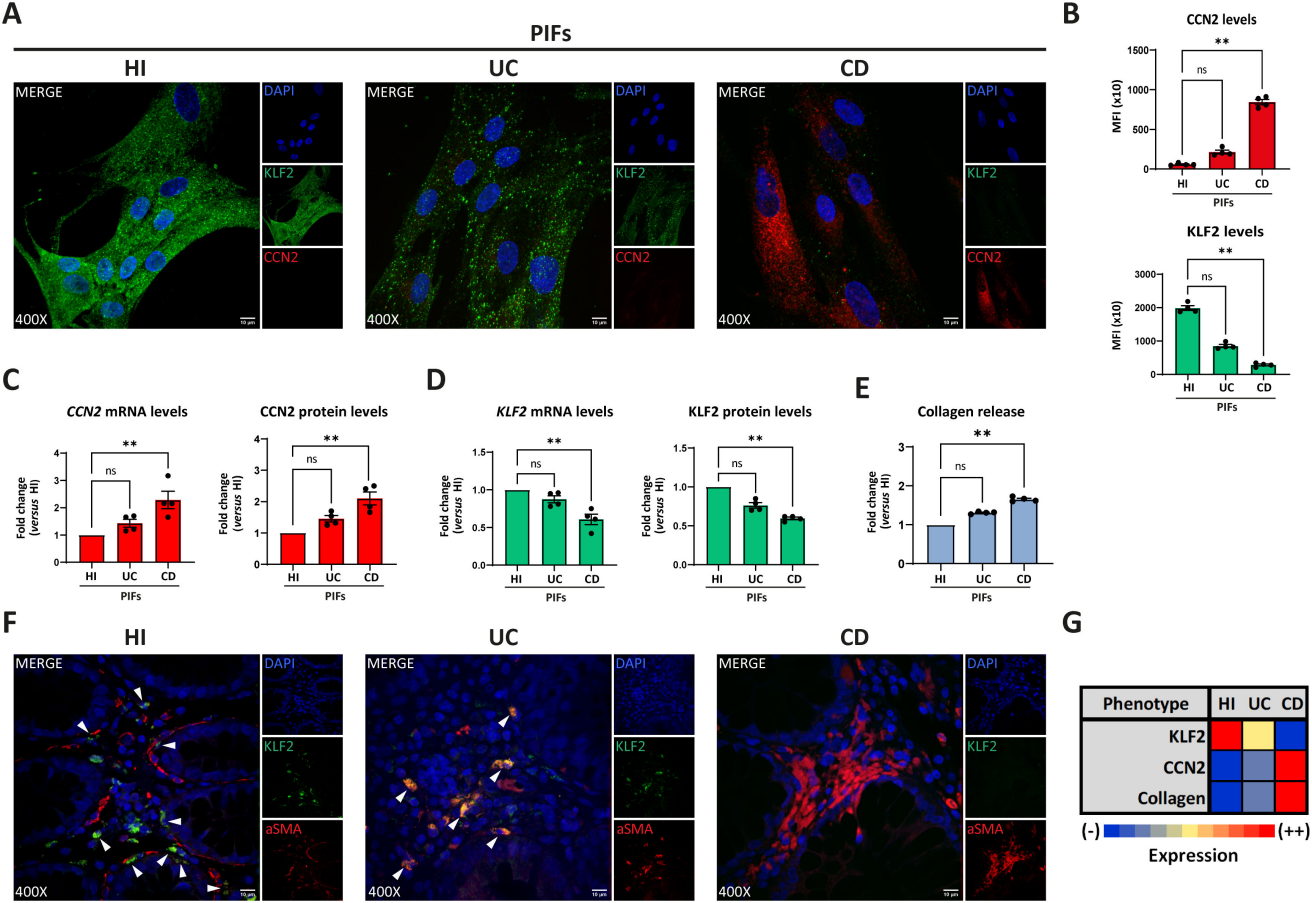
Crohn’s disease intestinal fibroblasts exhibit a distinct fibrotic phenotype. Assessment of KLF2 and CCN2 expression in PIFs from IBD patients and HI by **(A)** immunostaining (blue: DAPI, green: KLF2, red: CCN2), and **(B)** corresponding MFI quantification. **(C)** CCN2 and **(D)** KLF2 mRNA and protein levels assessed by RT-qPCR and in-cell ELISA, respectively. **(E)** Collagen release was measured in supernatants collected from the above-mentioned PIFs. **(F)** KLF2 expression in myofibroblasts within intestinal biopsies obtained from the same patients (blue: DAPI, green: KLF2, red: aSMA). **(G)** Heatmap indicating the expression intensity of KLF2, CCN2 and collagen release in PIFs. **(A, F)** One representative example out of four independent experiments, performed in different subjects of each group, is shown. **(F)** White arrowheads show double positive KLF2/aSMA cells observed in HI and UC patients. Confocal microscopy. **(A, F)** Magnification: 400x, Scale Bar: 10μm. Nonparametric Kruskal-Wallis followed by Dunn’s multiple comparisons test was applied in all panels, *n=4*, **p<0.01, ns, not significant. Data are expressed as mean ± SEM. aSMA, alpha smooth muscle actin; CCN2, cellular communication network factor 2; CD, Crohn’s disease; HI, healthy individuals; IBD, inflammatory bowel disease; KLF2, Kruppel-like factor 2; MFI, mean fluorescence intensity; PIFs, primary intestinal fibroblasts; UC, ulcerative colitis.

We studied the phenotype of cultured primary intestinal fibroblasts (PIFs), isolated from treatment-naïve patients with active CD and UC, as well as from HI. UC was included as a control inflammatory bowel, non-typical fibrotic, disease. In contrast to UC, CD PIFs, compared to HI PIFs, showed significantly lower KLF2 and higher CCN2 mRNA and protein levels (**Figures 1A–D**), as well as increased expression of alpha smooth muscle actin (aSMA) and collagen type 1 (COL1), as assessed by immunofluorescence (**Supplementary Figure S1A**). In line with these, CD PIFs were characterized by elevated collagen release (**Figure 1E**).

Next, we examined intestinal tissues obtained from the same patients. In agreement with our previous findings in isolated cultured PIFs, CD tissues displayed an abundance of aSMA-positive fibroblasts and enhanced COL1 production compared to UC and HI tissues (**Supplementary Figure S1B**). Moreover, in contrast to UC, aSMA-positive cells in CD tissues were characterized by enhanced CCN2 (**Supplementary Figure S2A**) and reduced KLF2 staining (**Figure 1F**; **Supplementary Figure S2B**). No differences were observed between CD tissue sections obtained from patients with inflammatory strictures (Montreal score B2) and those with non-stricturing, inflammatory behavior of the disease (Montreal score B1) (**Supplementary Figure S1B**). Similar to isolated PIFs, in tissues stained for vimentin, CD fibroblasts were also KLF2 negative, while UC and HI tissue fibroblasts exhibited faint and bright KLF2 staining, respectively (**Supplementary Figure S2C**).

Collectively, we observed a negative association between KLF2 and CCN2 in the fibroblasts of CD patients. CD fibroblasts expressed the highest mRNA and protein levels of CCN2 in association with the highest collagen production, whereas HI fibroblasts expressed the highest levels of KLF2 with concomitant absence of CCN2 expression and collagen production. Based on these findings, we suggested an arbitrary grading scale, according to the expression intensity of the abovementioned parameters (**Figure 1G**). CD PIFs demonstrated fibrotic activity and displayed a distinct phenotype, compared to UC PIFs. For the sake of brevity, we characterized the former as KLF2 (-), CCN2 (++), collagen (++) cells, in contrast to UC KLF2 (+), CCN2 (-), collagen (-) PIFs and HI KLF2 (++), CCN2 (-), collagen (-) PIFs (**Figure 1G**).

### 3.2 The fibroblast-derived IL-8 is associated with the presence of neutrophils in the intestine of patients with inflammatory bowel disease

**Figure 2:**
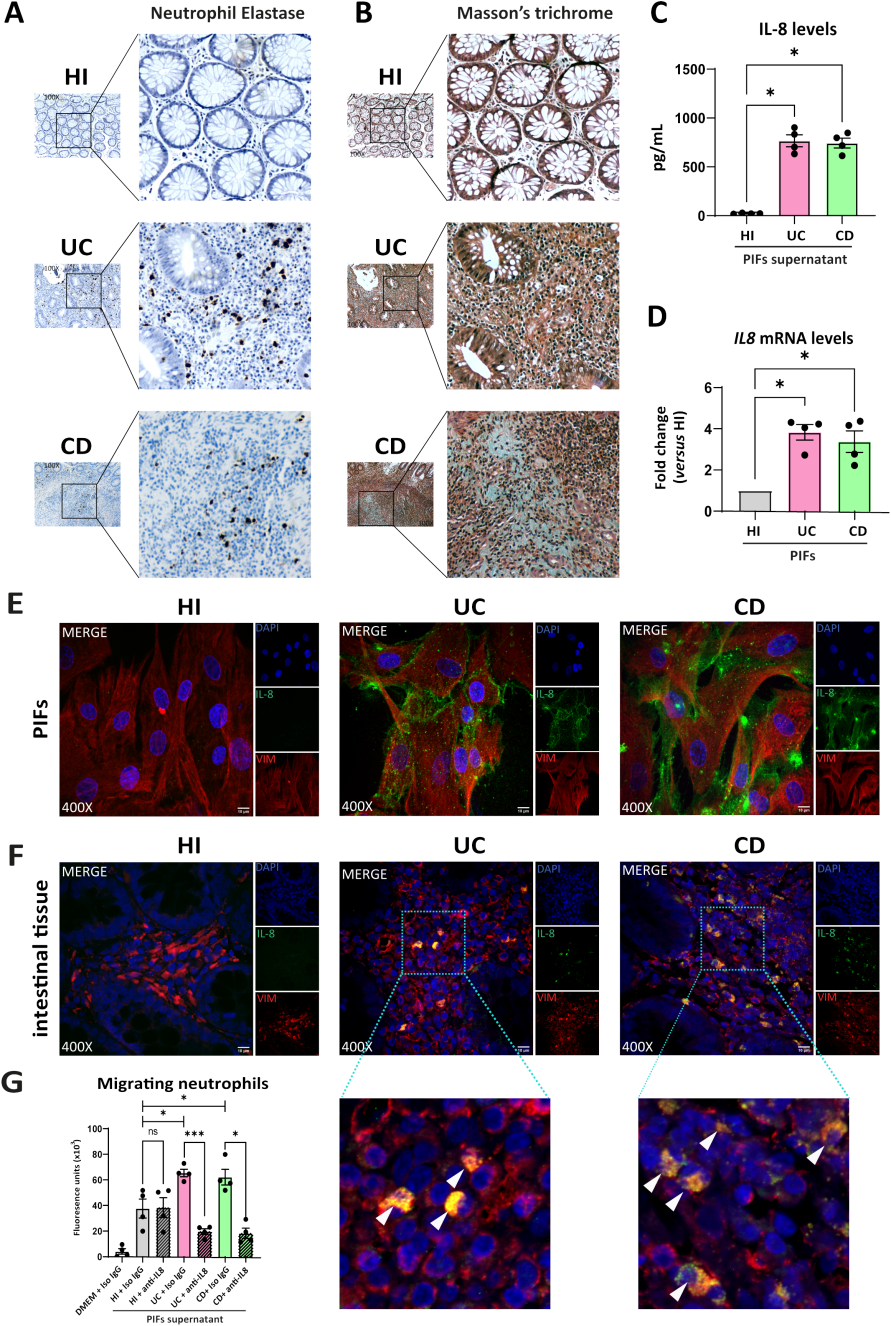
UC and CD intestinal tissues are characterized by differential spatial distribution of neutrophils, attracted in the intestinal tissue by fibroblast-derived IL-8. **(A)** Neutrophil Elastase IHC staining (brown cells) and **(B)** Masson’s trichrome (cyan fibers) indicating the presence of neutrophils and fibrotic areas respectively, in serial cross sections obtained from the same intestinal biopsies. Thickness between the serial cross sections in **(A, B)** was 4 μm. **(C)** IL-8 levels in supernatants of PIFs measured by a bead-based flow cytometric assay. **(D)** IL-8 mRNA assessed by RT-qPCR and **(E)** IL-8 protein levels in PIFs immunostaining (blue: DAPI, green: IL-8, red: Vimentin). **(F)** IL-8 expression assessed by immunostaining in intestinal tissue fibroblasts (blue: DAPI, green: IL-8, red: Vimentin). Dotted frames indicate the zoomed-in areas, which are provided to assess the co-expression of IL-8 and Vimentin. White arrowheads show double positive IL-8/Vimentin cells observed in UC and CD patients. **(G)** Chemotactic capacity of the PIFs’ supernatant on HI neutrophils, before and after the neutralization of IL-8, assessed by a transwell migration assay. **(A, B, E, F)** One representative example out of four independent experiments, performed in different subjects of each group, is shown. **(A, B)** Optical microscopy, magnification: 100x, **(E, F)** Confocal microscopy, magnification: 400x, Scale Bar: 10μm. Nonparametric Kruskal-Wallis followed by Dunn’s multiple comparisons test was performed in **(C, D)**, *n=4*, *p<0.05, ns, not significant. **(G)** Bayesian unpaired t-tests, followed by the Benjamini-Hochberg correction, were used to compare the migratory capacity of HI PIFs supernatants to UC and CD. For comparisons between PIFs supernatants that were treated with IL-8 neutralizing antibody (anti-IL-8), and supernatants treated with IgG isotype control (Iso IgG), Bayesian paired t-tests were performed, *n=4*, *p<0.05, ***p<0.001, ns, not significant. Data are expressed as mean ± SEM. CD, Crohn’s disease; DMEM, Dulbecco’s Modified Eagle Medium; HI, healthy individuals; IHC, immunohistochemistry; PIFs, primary intestinal fibroblasts; UC, ulcerative colitis;.

Since previous studies suggested that neutrophils may activate fibroblasts and interfere in the fibrotic process (10–12), we sought to investigate the distribution of neutrophils in intestinal tissues of active IBD, as well as their association with fibrotic lesions, in the same patients described above. Higher numbers of neutrophils were observed in UC, compared to CD intestinal tissues. (**Figure 2A**). However, neutrophils in intestinal sections of CD were found in the vicinity of the fibrotic areas, as defined by positive Masson’s trichrome staining for collagen, a pattern not observed in UC tissues (**Figures 2A, B**). As expected, neutrophils and fibrosis were absent from healthy intestinal tissues (**Figures 2A, B**).

Next, to decipher the mechanism by which neutrophils are attracted to the intestinal mucosa, we investigated whether fibroblasts produce known chemoattractant factors. Thus, we found that IL-8 (CXCL8) was the chemokine with significantly higher levels in supernatants of cultured PIFs obtained from UC and CD compared to HI (**Figure 2C**). Increased IL-8 expression was also observed in isolated PIFs both at mRNA (**Figure 2D**) and protein levels (**Figure 2E**), as well as in fibroblasts of intestinal sections from UC and CD patients, as assessed by IL-8/Vimentin (**Figure 2F**) and IL-8/aSMA (**Supplementary Figure S3**) staining. No significant alterations were observed in the levels of other cytokines in the PIFs’ supernatants obtained from IBD patients and HI (**Supplementary Figure S4**).

Prompted by these results and in view that IL-8 has a distinct target specificity for neutrophils (37), we next performed a chemotactic assay that indicated increased migratory capacity of neutrophils when stimulated by the IL-8-rich supernatant of UC and CD PIFs, an effect that was inhibited after IL-8 neutralization (**Figure 2G**).

These data suggest that both UC and CD fibroblasts express functional IL-8 as a key neutrophil chemoattractant factor in the intestinal environment. However, it warrants further investigation if neutrophils in each disease have differential functional properties during their crosstalk with fibroblasts.

### 3.3 *Ex-vivo* enriched neutrophil extracellular traps from CD patients induce a CD-like fibrotic phenotype in healthy PIFs

To investigate the hypothesis that neutrophils of CD, UC patients, or HI have distinct functional properties in their crosstalk with fibroblasts, we obtained mixtures consisting of *ex-vivo* NETs enriched with the supernatant formed during the isolation procedure of NETs, to preserve the total inflammatory environment including structures of DNA scaffold and cellular extracts (eNETs). Moreover, eNETs were collected after *in-vitro* stimulation of HI neutrophils with PMA, a chemical inducer of NETosis, and used as a non-disease-specific stimulus.

**Figure 3:**
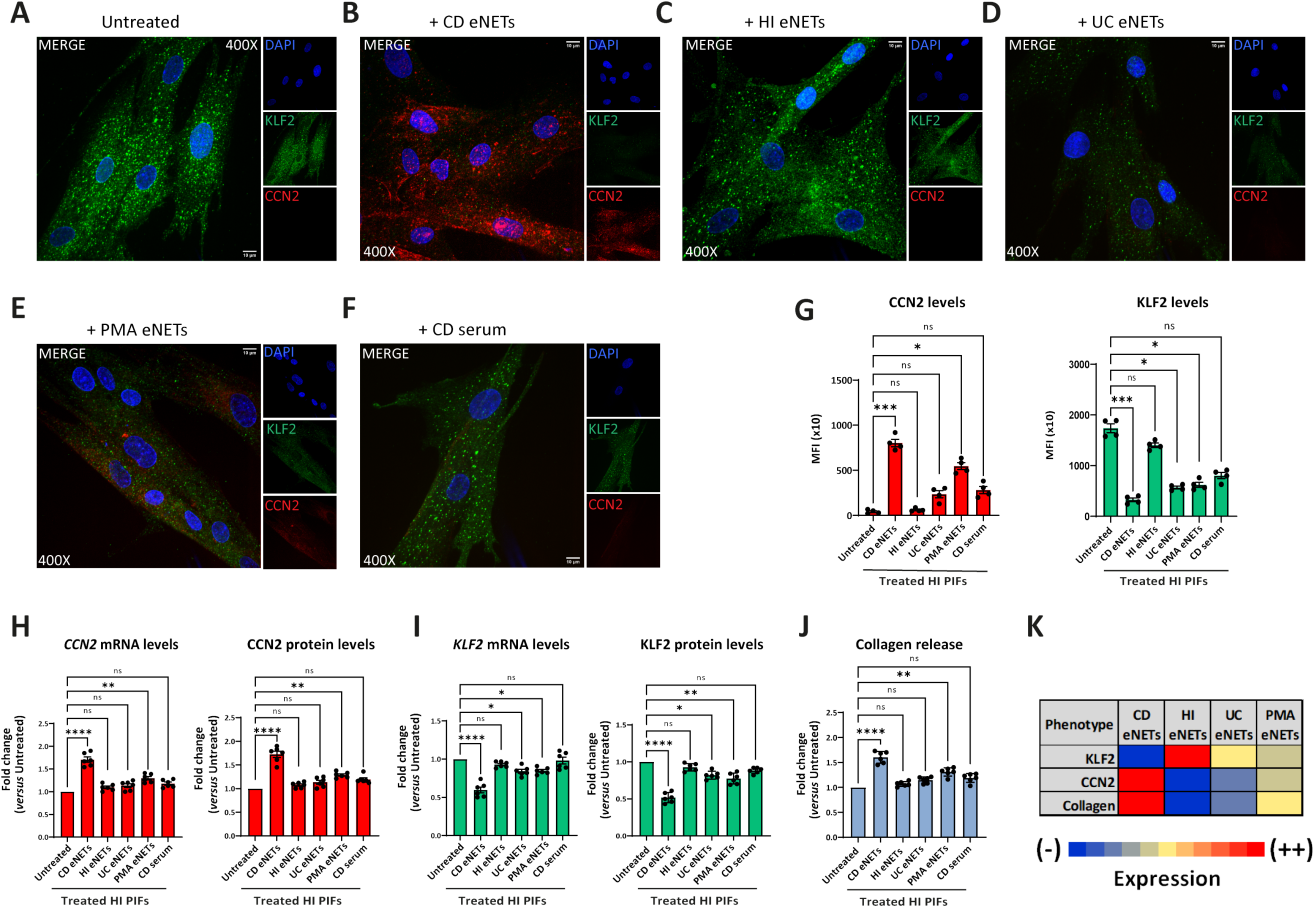
Treatment of healthy PIFs with *ex-vivo* isolated CD eNETs induces a CD-like fibrotic phenotype. **(A–F)** Immunostaining (blue: DAPI, green: KLF2, red: CCN2) and **(G)** corresponding MFI quantification of HI PIFs treated with *ex-vivo* isolated eNETs from **(B)** CD, **(C)** HI, or **(D)** UC patients, **(E)** PMA-generated eNETs and **(F)** CD serum. Assessment of CCN2 **(H)** and KLF2 **(I)** mRNA and protein levels by RT-qPCR and in-cell ELISA, respectively, as well as collagen release **(J)** in the treated PIFs. **(K)** Heatmap depicting the expression intensity of KLF2, CCN2 and collagen release. **(A–F)** One representative example out of four independent experiments is shown. Confocal microscopy. Magnification: 400x, Scale Bar: 10μm. **(G–J)** Nonparametric Kruskal-Wallis followed by Dunn’s multiple comparisons test, **(G)** *n=4*, **(H–J)** *n=6*, *p<0.05, **p<0.01, ***p<0.001, ****p<0.0001, ns, not significant. Data are expressed as mean ± SEM. CCN2, cellular communication network factor 2; CD, Crohn’s disease; eNETs, enriched neutrophil extracellular traps; HI, healthy individuals; KLF2, Kruppel-like factor 2; MFI, mean fluorescence intensity; PIFs, primary intestinal fibroblasts; PMA, Phorbol-12-myristate-13-acetate; UC, ulcerative colitis.

Next, we used these neutrophilic mixtures for stimulations on HI PIFs to assess KLF2 and CCN2 mRNA and protein levels, as well as collagen release. We found that HI PIFs stimulated with CD eNETs acquired a CD-like KLF2 (-), CCN2 (++), and collagen (++) fibrotic phenotype (**Figures 3A, B, G–K**). This effect was not observed in stimulations with HI eNETs (**Figures 3C, G–K**). In contrast, UC eNETs drove fibroblasts towards the UC-like phenotype, indicating KLF2 (+), CCN2 (-), and collagen (-) cells (**Figures 3D, G–K**). Moreover, PMA-generated mixtures led fibroblasts towards KLF2 (+), CCN2 (+), and collagen (+) phenotype, indicative of a mild fibrotic activity (**Figures 3E, G–K**). The fact that the serum of CD patients alone was unable to transform the phenotype of HI fibroblasts towards acquiring fibrotic function further supports the key role of neutrophils in the activation of fibroblasts (**Figures 3F–J**).

Taken together, CD neutrophils probably acquire different plasticity compared to UC neutrophils, being able to transform healthy PIFs into a CD-like fibrotic phenotype, demonstrating a functional role in their crosstalk with intestinal fibroblasts.

### 3.4 Transcriptome analysis of peripheral blood neutrophils unravels distinct pathways in Crohn’s disease and ulcerative colitis

**Figure 4:**
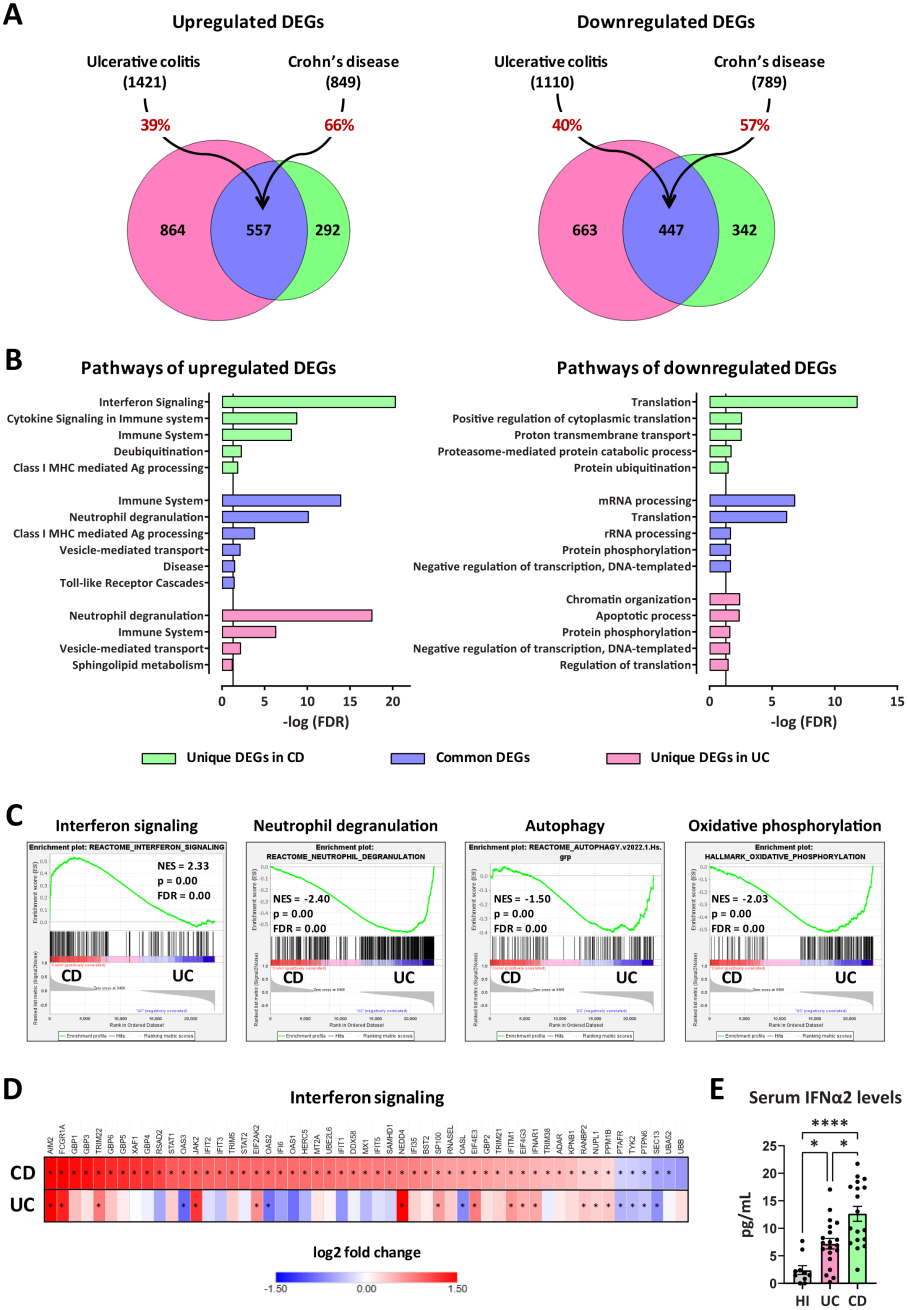
Transcriptomic analysis of peripheral blood neutrophils isolated from patients with CD or UC. **(A)** Venn diagrams showing upregulated and downregulated DEGs (baseMean > 30 and FDR < 0.05), following RNA-Seq analysis of peripheral blood neutrophils isolated from patients with CD (*n=18*) or UC (*n=24*). DEGs were identified following comparison with neutrophils isolated from healthy individuals (*n=18*). The percentages on the arrows indicate the overlap between the two diseases. **(B)** Graphs depicting the top upregulated and downregulated pathways in neutrophils isolated from CD or UC patients. Reactome and GO Biological Process annotations were used for up- and down-regulated DEGs, respectively. Pathways with redundant sets of DEGs were excluded. Vertical lines show the threshold for statistical significance (FDR < 0.05). **(C)** GSEA plots of significantly altered signatures in the transcriptome of CD *versus* UC neutrophils, using Reactome and Hallmark as reference gene sets from the Human Molecular Signatures Database. **(D)** Heatmap depicting the log2 fold change values of DEGs belonging to the Interferon signaling pathway (Reactome, R-HSA-913531.3), as determined by RNA-Seq analysis of CD and UC neutrophils. Asterisks depict statistical significance (FDR < 0.05). **(E)** Levels of IFNα2 in the serum of HI and patients with CD or UC. Data are expressed as mean ± SEM. Nonparametric Kruskal-Wallis test was applied, followed by Dunn’s multiple comparisons test, *p < 0.05, and ****p < 0.0001. CD, Crohn’s disease; DEGs, differentially expressed genes; FDR, false discovery rate; GSEA, gene set enrichment analysis; HI, healthy individuals; NES, normalized enrichment score; RNA-Seq, RNA-Sequencing; UC, ulcerative colitis.

To elucidate the mechanistic differences underlying the plasticity observed in IBD neutrophils, we sought to compare the transcriptome of peripheral blood neutrophils isolated from CD and UC patients. Our analysis identified 849 significantly upregulated and 789 downregulated genes in CD neutrophils, whereas 1421 genes were found upregulated and 1110 downregulated in UC neutrophils, compared to control neutrophils purified from healthy individuals (**Figure 4A**). Interestingly, the two datasets exhibited a substantial overlap since 66% of upregulated and 57% of downregulated genes in CD were also significantly regulated in UC neutrophils (**Figure 4A**; **Supplementary Figure S5**). Bioinformatics analysis using the GeneCodis4 web-based tool revealed that common upregulated DEGs clustered mainly in immune-related pathways, including neutrophil degranulation, class I MHC mediated antigen processing and presentation, and Toll-like receptor cascades, while translation, mRNA and rRNA processing were amongst the top downregulated processes (**Figure 4B**). Apart from the commonly regulated DEGs and pathways, we found several genes and processes uniquely regulated in each disease (**Figure 4B**; **Supplementary Figure S5**). More specifically, interferon signaling was the top pathway selectively upregulated in Crohn’s (**Figure 4B**), while the majority of unique upregulated genes in UC were involved in the neutrophil degranulation pathway. Regarding the unique downregulated genes, these are clustered mainly to translation- and post-translational modification-related pathways in CD neutrophils, while the respective DEGs in UC neutrophils belonged to the pathways of chromatin organization and apoptotic process among others (**Figure 4B**).

Gene set enrichment analysis (GSEA) was also performed, using the Reactome and Hallmark gene sets collections of the Human Molecular Signatures Database, to reveal enriched signatures in our datasets, and independently verify the overrepresentation of interferon signaling and neutrophil degranulation in CD and UC neutrophils, respectively (**Figure 4C**). GSEA also highlighted the increased expression of DEGs involved in autophagy and oxidative phosphorylation in UC, compared to CD neutrophils, consistent with the previously reported increased NETotic potential in UC (20).

Focusing on the interferon signaling pathway, we identified 53 DEGs significantly regulated in CD neutrophils, including several target genes, intracellular mediators, enzymes, and receptors (**Figure 4D**). Of these, 47 were up- and only six were down-regulated. On the other hand, only 14 out of 53 aforementioned genes were significantly upregulated in UC, while three major interferon-induced genes (*OAS2*, *OAS3*, and *OASL*) were downregulated (**Figure 4D**; **Supplementary Figure S5E**). Moreover, RT-qPCR analysis in neutrophils from an independent cohort of CD and UC patients confirmed that mRNA levels of the key IFN signaling genes *STAT1* and *STAT2* followed the same expression pattern with transcriptome analysis (**Supplementary Figure S6A**). Hence, the molecular signature that distinguishes CD from UC neutrophils is positively correlated with interferon signaling activation.

Next, we sought to measure the levels of interferons, along with several other cytokines in the sera of patients with CD and UC. Although both IFNα2 and IFNγ were found elevated in the sera of IBD patients, in comparison to healthy individuals, only IFNα2 levels were significantly higher in CD over UC patients (**Figure 4E**; **Supplementary Figure S6B**). Thus, elevated IFNα2 levels in the serum possibly account for the interferon fingerprint characterizing the transcriptome of peripheral neutrophils in CD.

### 3.5 IFNα primes neutrophils of CD patients to acquire fibrotic partnership

Based on the findings above, we hypothesized that IFNα may signal in neutrophils committing them to acquire a fibrotic partnership in their crosstalk with fibroblasts. To this end, we stimulated *in-vitro* HI neutrophils with serum from CD patients to produce eNETs (CD serum-generated eNETs), which were subsequently used to treat HI primary fibroblasts.

**Figure 5:**
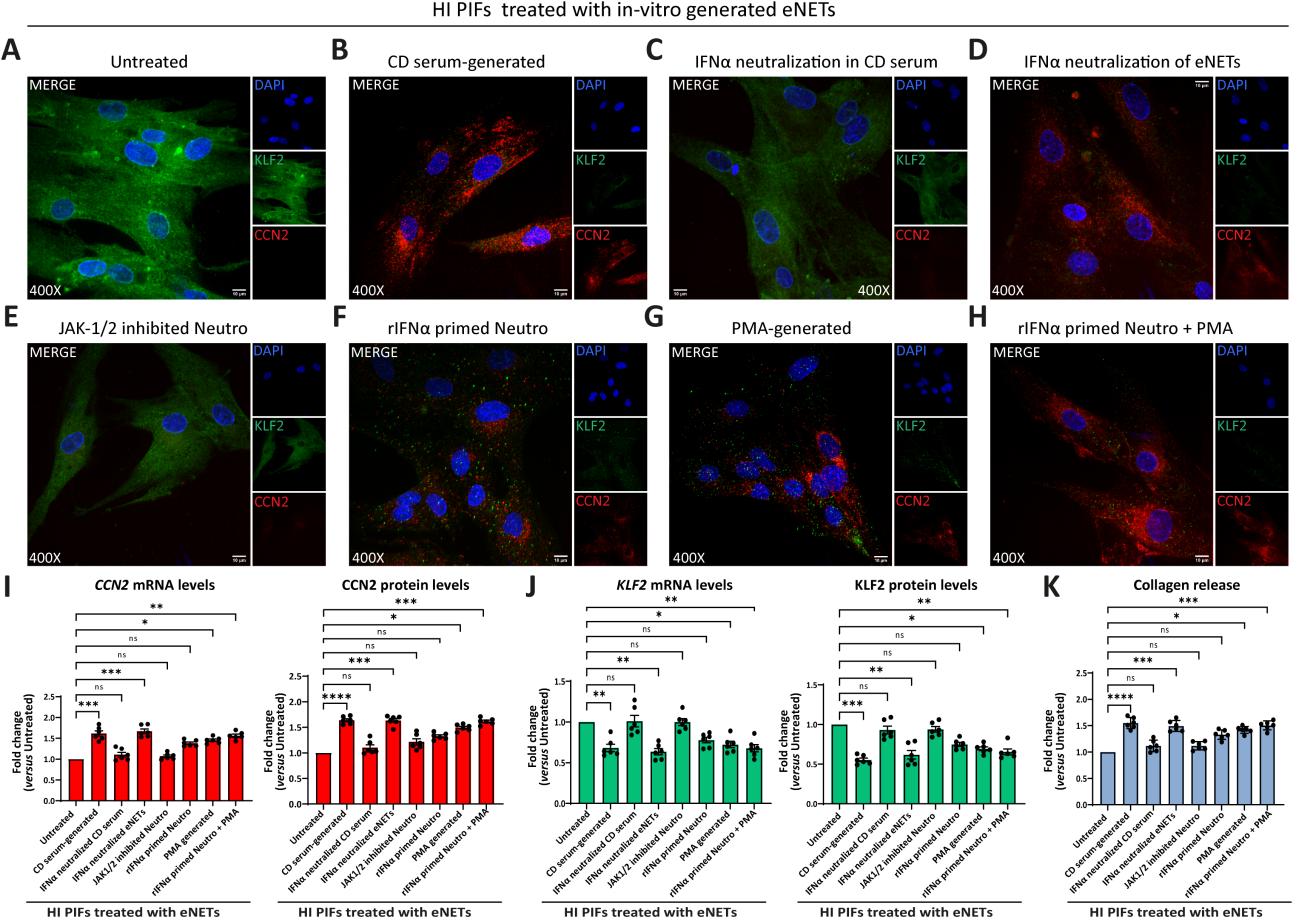
CD neutrophils are primed through IFNα/JAK signaling to exert their fibrotic role. **(A-H)** Evaluation of CCN2 and KLF2 by immunofluorescence (blue: DAPI, green: KLF2, red: CCN2) in HI PIFs treated with *in-vitro* generated eNETs under various conditions: **(A)** HI neutrophils stimulated by CD serum to form eNETs in the **(B)** absence or **(C)** presence of an IFNα neutralizing antibody. **(D)** IFNα neutralization of already formed CD-serum eNETs. **(E)** Inhibition of JAK-1/2 signaling in neutrophils with baricitinib. **(F)** Neutrophils primed with IFNα to produce eNETs. **(G)** PMA-generated eNETs. **(H)** Combination of **(F)** and **(G)** to produce eNETs that subsequently treated HI PIFs. **(I)** CCN2 and **(J)** KLF2 mRNA and protein levels in eNETs-treated fibroblasts, as assessed by RT-qPCR and in-cell ELISA. **(K)** Corresponding collagen release assay. **(A–H)** One representative example out of four independent experiments is shown. Confocal microscopy. Magnification: 400x, Scale Bar: 10μm **(I–K)** Nonparametric Kruskal-Wallis followed by Dunn’s multiple comparisons test, *n=6*, *p<0.05, **p<0.01, ***p<0.001, ****p<0.0001, ns, not significant. Data are expressed as mean ± SEM. CCN2, cellular communication network factor 2; CD, Crohn’s disease; eNETs, enriched neutrophil extracellular traps; HI, healthy individuals; JAK, Janus kinase; KLF2, Kruppel-like factor 2; MFI, mean fluorescence intensity; Neutro, neutrophils; PIFs, primary intestinal fibroblasts; PMA, Phorbol-12-myristate-13-acetate; rIFNα, recombinant interferon alpha.

Our results showed that fibroblasts treated with CD serum-generated eNETs acquired a fibrotic phenotype; KLF2 (-), CCN2 (++), and collagen (++) (**Figures 5A, B, I-K**; **Supplementary Figures S7A, B**). This fibrotic transformation was not observed when we used CD serum-generated eNETs obtained after neutralizing IFNα in the serum (**Figures 5C, I–K**; **Supplementary Figures S7A, B**), or when HI neutrophils were treated with the JAK-1/2 inhibitor baricitinib before their stimulation by CD serum to form eNETs (**Figure 5E, I-K**; **Supplementary Figures S7A, B**). Neutralization of IFNα in the already formed CD serum-generated eNETs was ineffective in preventing fibroblasts from acquiring a fibrotic phenotype, suggesting that IFNα exerts an early, priming effect on neutrophils (**Figures 5D, I–K**; **Supplementary Figures S7A, B**).

Furthermore, healthy fibroblasts that initially acquired a KLF2 (+), CCN2 (+), collagen (+) mild fibrotic phenotype upon treatment with PMA-generated eNETs, were transformed towards KLF2 (-), CCN2 (++), collagen (++) fibrotic active cells, when recombinant IFNα primed the neutrophils during the procedure of PMA eNETs generation (**Figures 5F–K**; **Supplementary Figures S7A, B**).

Collectively, our data so far suggest that in CD, priming of circulating neutrophils by IFNα is essential to commit their fibrotic effect over intestinal fibroblasts and their plasticity is expressed through eNETs.

### 3.6 Neutrophil-dependent fibrosis in CD is eliminated by dismantling the DNA scaffold of eNETs or by inhibiting JAK signaling in fibroblasts

Prompted by the above-mentioned findings indicating that IFNα primes neutrophils to acquire a fibrotic role through eNETs and since previous results showed that JAK signaling in fibroblasts is involved in COVID-19 immunofibrosis (13), we tried to diminish the fibrotic transformation of fibroblasts either by dismantling the DNA scaffold of eNETs or by inhibiting JAK signaling in these cells.

**Figure 6:**
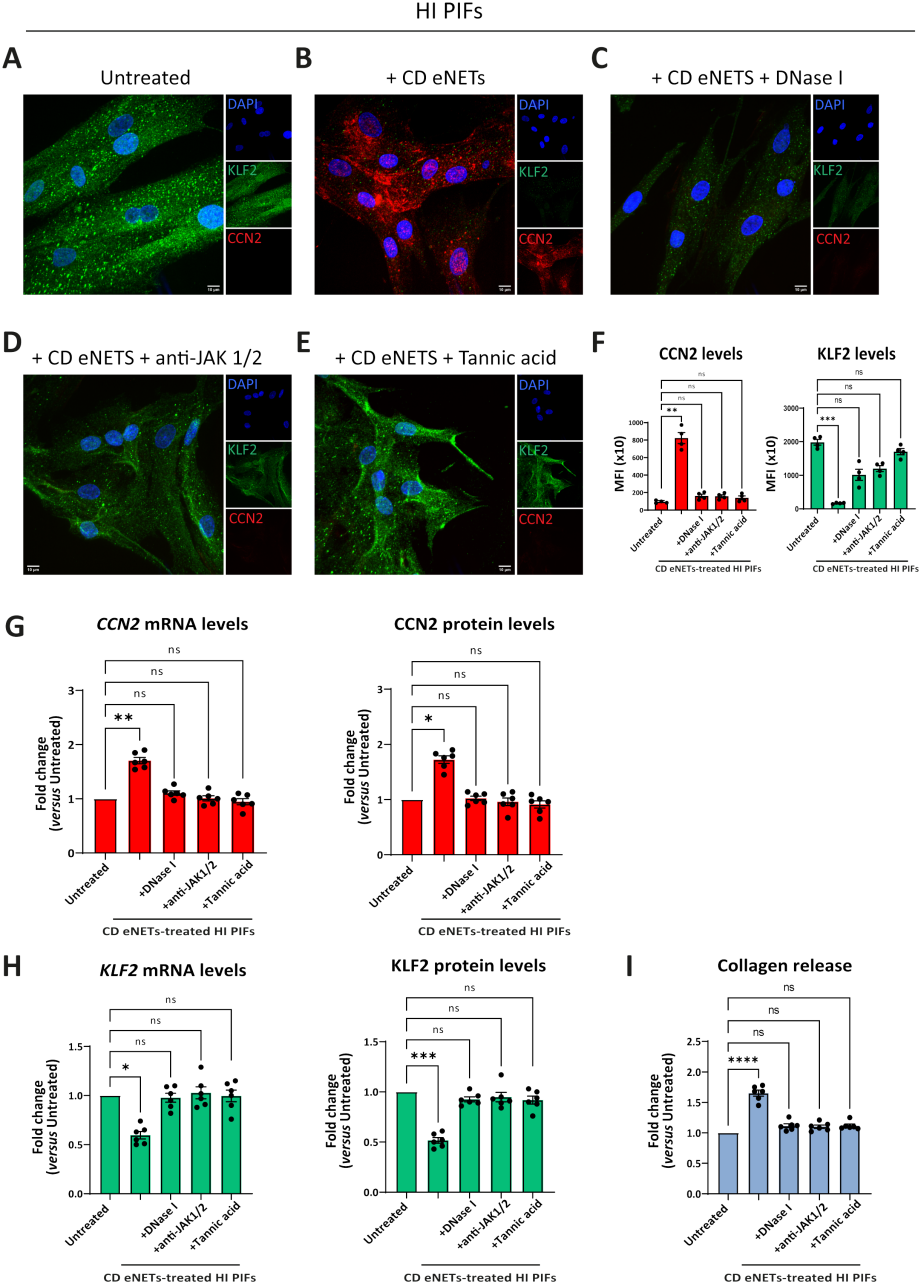
Disruption of NET-scaffold or inhibition of JAK-1/2 signaling in fibroblasts prevents neutrophil-mediated fibrosis. **(A–E)** Assessment of CCN2 and KLF2 by immunofluorescence (blue: DAPI, green: KLF2, red: CCN2) and **(F)** MFI quantification, in stimulation and inhibition studies. **(A)** HI PIFs, treated with **(B)** *ex-vivo* CD eNETs or **(C)** CD eNETs pre-treated with DNase I to dismantle the DNA-scaffold. **(D)** HI PIFs pre-treated with JAK-1/2 inhibitor baricitinib and subsequent stimulation with CD eNETs. **(E)** Tannic acid, a chemical inducer of KLF2, was used as a positive control of KLF2 expression. **(G)** Analysis of CCN2 and **(H)** KLF2 mRNA and protein expression, and **(I)** Collagen release assay, in PIFs from the abovementioned *in-vitro* studies. **(A–E)** One representative example out of four independent experiments is shown. Confocal microscopy. Magnification: 400x, Scale Bar: 10μm. **(F-I)** Nonparametric Kruskal-Wallis followed by Dunn’s multiple comparisons test, *n=6*, *p<0.05, **p<0.01, ***p<0.001, ****p<0.0001, ns, not significant. Data are expressed as mean ± SEM. CCN2, cellular communication network factor 2; CD, Crohn’s disease; eNETs, enriched neutrophil extracellular traps; HI, healthy individual; JAK, Janus kinase; KLF2, Kruppel-like factor 2; MFI, mean fluorescence intensity; STAT, signal transducer and activator of transcription.

Treatment of CD eNETs with DNase I or pretreatment of HI fibroblasts with baricitinib inhibited the transformation of the KLF2 (++), CCN2 (-), collagen (-) HI fibroblasts towards the KLF2 (-), CCN2 (++), collagen (++) CD-like phenotype (**Figures 6A–D, F–I**). This effect was also observed with the plant-derived polyphenol tannic acid, a potent inducer of KLF2 expression (13, 38), suggesting a potential role of KLF2 in the fibrotic process (**Figures 6E–I**). Next, we investigated whether JAK inhibition could reverse to normal the fibrotic phenotype of the already affected primary fibroblasts of CD. Following their treatment with baricitinib, the KLF2 (-), CCN2 (++), collagen (++) phenotype of active CD primary fibroblasts was not altered, suggesting that JAK inhibition should be performed early, before acquiring the CD phenotype (**Supplementary Figures S8A–E**).

In conclusion, neutrophil-fibroblast crosstalk in CD is disrupted by targeting neutrophil DNA scaffold or/and JAK signaling in fibroblasts before their interaction. This neutrophil-driven fibrotic process seems to be an early event in the pathogenesis of CD immunofibrosis.

### 3.7 Levels of interferon signaling components are positively correlated with CD severity

To further support our findings, we sought to examine whether serum IFNα levels and transcriptomic alterations in IFN signaling-related genes in peripheral neutrophils are associated with the severity of CD, as reflected by the classical disease activity index, CDAI (18).

**Figure 7:**
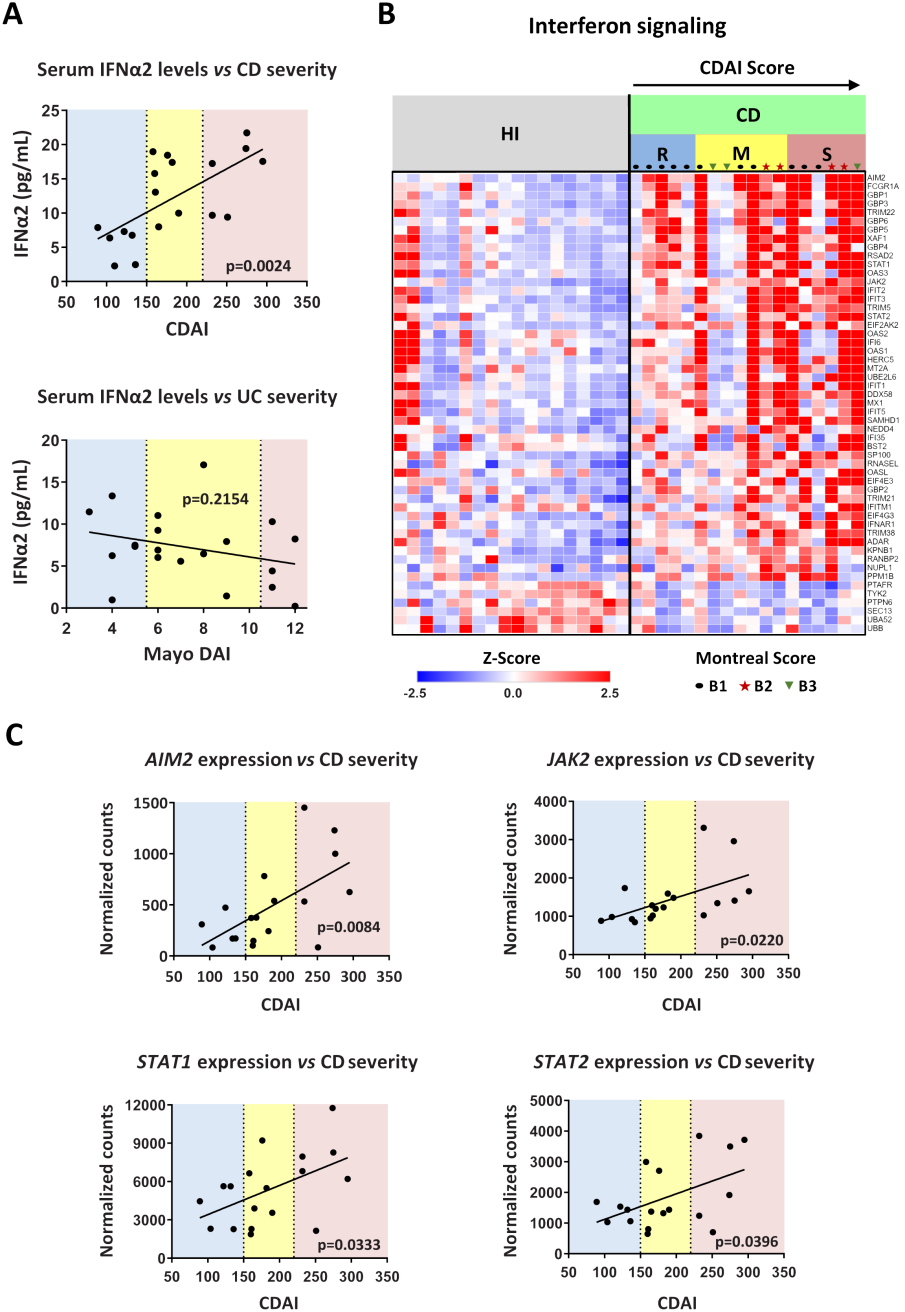
Levels of interferon signaling components are positively correlated with Crohn’s disease severity. **(A)** Correlation plots of serum IFNα2 levels *versus* disease severity in CD (upper graph) and UC (lower graph). CDAI and Mayo DAI were used to assess disease activity in CD and UC, respectively. **(B)** Heatmap depicting the relative expression of DEGs belonging to the Interferon signaling pathway (Reactome, R-HSA-913531.3), as determined by RNA-Seq analysis of neutrophils isolated from HI (*n=18*) and CD patients (*n=18*). **(C)** Correlation plots of the mRNA expression of key interferon signaling components (*AIM2*, *JAK2*, *STAT1*, and *STAT2*), as determined by RNA-Seq analysis of CD neutrophils, *versus* disease severity in CD. Simple linear regression was used in all panels to assess the relationship between the studied variables. CD, Crohn’s disease; CDAI, Crohn’s disease activity index (R, remission, < 150; M, mild to moderate, 150-220; S, moderate to severe, > 220); Mayo DAI, Mayo score disease activity index (mild, 3-5; moderate, 6-10; severe, 11-12); Montreal score according to disease behavior: B1, non-stricturing, non-penetrating (n=11); B2, stricturing (inflammatory strictures, n=4); B3, penetrating (n=3); HI, healthy individuals; RNA-Seq, RNA-Sequencing; UC, ulcerative colitis.

We observed that IFNα2 levels in the serum of CD patients were correlated with the disease severity, since individuals with higher CDAI scores were also characterized by higher concentrations of IFNα2 (**Figure 7A**). In contrast to CD, no such correlation was observed in UC between IFNα levels and Mayo DAI score, a common indicator of disease activity (17) (**Figure 7A**).

We also noticed that the transcriptomic alterations observed in peripheral blood neutrophils of IBD patients were also correlated with the CDAI and Mayo DAI scores (**Supplementary Figures S9A, B**). The whole set of DEGs followed an expression pattern related to the disease severity (**Supplementary Figures S9A, B**), an observation that was prominent for interferon signaling components in CD as well (**Figure 7B**). Specifically, the mRNA expression of key components of the interferon signaling pathway, namely *AIM2*, *JAK2*, *STAT1*, and *STAT2*, correlated closely with the CDAI score, increasing in line with disease severity (**Figure 7C**). Moreover, the expression of several interferon signaling components also correlated with serum IFNα2 levels (**Supplementary Figure S9C**). Although there was a trend toward higher IFNα2 levels in patients with inflammatory strictures (Montreal score B2), these levels did not differ significantly from those in patients with non-stricturing, non-penetrating disease (Montreal score B1) or penetrating disease (Montreal score B3) (**Supplementary Figure S10A**). Similarly, activation of IFNα signaling was independent of disease behavior as assessed by Montreal score (**Figure 7B**; **Supplementary Figure S10B**). This suggests that IFNα pathway is implicated in CD inflammatory environment that may lead to various disease complications, including strictures.

Taken together, serum IFNα and/or mRNA levels of interferon signaling components in neutrophils emerge as potential diagnostic/prognostic biomarkers for CD.

## 4 Discussion

In this study, we describe a novel mechanistic link between neutrophils and fibroblasts that characterizes immunofibrosis of CD, as a precursor of a putative, later stricturing disease. In contrast to UC, neutrophils of CD patients, exhibit pro-fibrotic properties by activating intestinal fibroblasts leading to increased collagen release. This type of neutrophil plasticity is primed by IFNα signaling and expressed by the NET-enriched inflammatory environment. Moreover, neutrophils may be attracted to the intestinal mucosa by IL-8 secreted from activated fibroblasts sustaining this fibrotic loop of neutrophil-fibroblast interaction. Further supporting this mechanistic basis, the expression levels of key IFNα pathway components in serum and neutrophils are well correlated with the clinical activity of CD patients.

Growing evidence today implies that the development of tissue fibrosis involves the complex interplay between immune and stromal cells. However, in CD, cell-cell interactions and functions are incompletely understood, with much research on lymphocytes and/or other mononuclear immune cells, while the role of neutrophils remains obscure (14).

Here, we showed that neutrophils in active CD and UC may be recruited at the site of tissue damage by IL-8 produced by intestinal fibroblasts. Earlier clinical studies have indicated increased IL-8 levels in the intestinal mucosa of active IBD patients (39, 40). Our study further characterizes fibroblasts as a source of IL-8 in the tissue environment of IBDs suggesting a functional, chemoattractant, effect on peripheral neutrophils. Future studies should aim to elucidate the specific mediator(s) responsible for initiating IL-8 expression in fibroblasts, as well as explore the putative role of gut microbiota dysbiosis. Similar triggers might also induce IL-8 production by connective tissue mesenchymal cells in IBD extraintestinal manifestations, such as arthritis. In support of this, it has been previously shown that NETs from rheumatoid arthritis patients can stimulate the production of IL-8 from fibroblast-like synoviocytes (10). Of note, although fibroblast-derived IL-8 appears to be implicated in neutrophil recruitment in both IBDs, we observed a differential intestinal distribution of neutrophils. In CD, dense neutrophil infiltrations were observed in proximity to fibrotic areas, implying a fibrotic role for recruited neutrophils. This finding combined with the differences in the plasticity of neutrophils between the two IBDs could explain the fibrotic phenotype of CD.

Currently, the traditional concept that neutrophils comprise terminally differentiated cells with limited plasticity and highly conserved function has been critically revised (5, 6). Several studies now support that during inflammatory conditions, the transition of mature neutrophils from bone marrow to the bloodstream is accompanied by changes at the transcriptional level that enable the acquisition of distinct functions within the affected tissues (5, 6). Previous clinical and experimental studies suggest that in the context of different inflammatory disorders, neutrophils may acquire differential plasticity which may be reflected by the NETs that they release (9, 10). In this context, our group and others have indicated that activated neutrophils may acquire an immunofibrotic role through the release of NETs. These fibrogenic NETs are able to activate human fibroblasts, inducing their proliferation, differentiation to myofibroblasts and immunogenicity (10–12).

Our functional studies indicated that NET-enriched extracellular mediators (eNETs) *ex-vivo* isolated from peripheral neutrophils of CD patients, and not the CD serum directly, were able to transform healthy intestinal fibroblasts toward the distinct CD phenotype characterized by negative KLF2 and high CCN2 expression, leading to collagen production. A similar immunofibrotic phenotype of lung fibroblasts has been also described in severe COVID-19 (13). However, eNETs from peripheral neutrophils of UC patients did not exhibit a similar potential, suggesting differential neutrophil/NETs plasticity on fibroblasts between the two IBDs.

To identify possible molecular pathways and targets that may drive this plasticity, we applied whole transcriptome analysis in peripheral blood neutrophils from IBD patients. Importantly, although a substantial overlap of the DEGs between the two intestinal diseases was observed, peripheral neutrophils of CD were selectively characterized by an IFN-responsive signature. In contrast, UC neutrophils showed upregulation in genes and pathways related to neutrophil degranulation, autophagy and oxidative phosphorylation, confirming our previous mechanistic studies which demonstrated the key role of autophagy-mediated NET formation in UC (20). Recently, top-upregulated severity genes in the colonic mucosa of UC patients have been also found to be involved in innate immunity and neutrophil degranulation (41). Previous comparative transcriptome studies between UC and CD have yielded heterogeneous results mostly being non-targeted, performed in whole blood or mucosal tissue (42–44). Despite this, a mostly neutrophil-like signature has been proposed for the whole blood of IBD patients, while the most significant signal within CD ileal mucosa with deep ulcers was for granulocytes, further favoring the role of neutrophils in active IBD (42, 44). At the time of this writing, emerging single-cell transcriptome data in mucosal samples reveal the heterogeneity of intestinal neutrophils in IBD patients. According to A. Garrido-Trigo et al., intestinal neutrophils exist in three distinct states—N1, N2, and N3—whose relative abundance varies by individual patient and disease type (45). UC patients exhibit a higher abundance of N1 neutrophils (~65% in UC vs. ~20% in CD), while CD patients are characterized by a higher prevalence of N3 neutrophils (~70% in CD vs. ~20% in UC). Our data reveal that peripheral UC neutrophils differentially express genes associated with the N1 and N2 intestinal populations, including those related to the neutrophil degranulation pathway. In contrast, peripheral CD neutrophils overexpress genes related to the N3 signature of intestinal neutrophils. Notably, N3 intestinal neutrophils display a marked IFN-response signature, which is also evident in CD peripheral neutrophils. Commonly overexpressed IFN-response transcripts include *GBP1, GBP2, GBP4, GBP5, IFIT2, IFIT3*, and *RSAD2* (**Supplementary Figure S11**). Therefore, at least the interferon fingerprint is maintained after neutrophil migration into the intestinal tissue.

Based on the findings indicating that IFN signature may distinguish CD from UC neutrophils, and multiplex cytokine analysis showing that IFNα, and not IFNγ, serum levels were significantly increased in CD compared to UC, it was reasonable to assume that IFNα may prime peripheral neutrophils of CD patients to exert their fibrotic effect on fibroblasts. In line with this, functional studies indicated that CD serum induces the generation of highly fibrogenic eNETs in an IFNα-dependent manner. Furthermore, neutrophils primed with IFNα, during the production of PMA-generated eNETs, enhanced their fibrotic plasticity. This further supports an additional key role for IFNα in the immunofibrotic plasticity of neutrophils, which is expressed *via* extracellular DNA structures. A large body of evidence has demonstrated that the integrity of DNA scaffold is important for NETs function (10, 11, 46). How the architecture of NETs and their associated proteins are implicated in the immunofibrotic plasticity of neutrophils is an intriguing question warranting further investigation in the future.

Several studies have indicated that loss-of-function mutations in nucleotide-binding oligomerization domain 2 (NOD2) have been associated with CD (47). Of interest, it has been recently indicated that NOD2 activation in hematopoietic cells protected mice from TLR9-induced exacerbation of DSS-induced colitis by downregulating IFNα responses (48). Although several immune and non-immune cells may contribute, the exact sources of IFNα in the systemic circulation of CD remain to be identified. It has been previously shown that mature neutrophils in systemic lupus erythematosus (SLE) are primed *in-vivo* by type I IFNs to release NETs upon exposure to SLE-derived autoantibodies, indicating plasmacytoid dendritic cells as a major source of IFNα (49).

**Figure 8:**
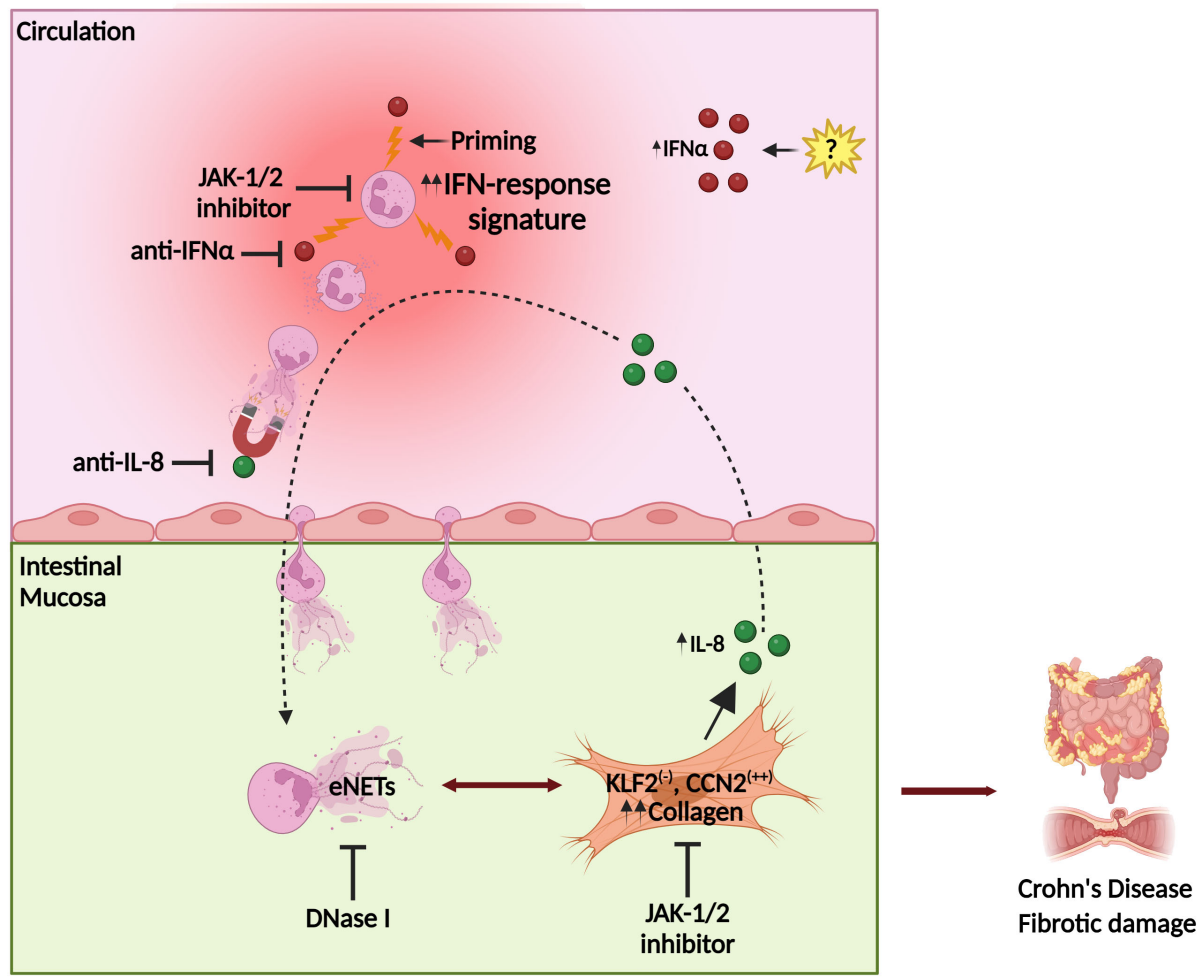
Proposed model of Crohn’s disease immunofibrosis mediated by neutrophil-fibroblast crosstalk. Peripheral neutrophils are primed by IFNα, acquiring pro-fibrotic plasticity. Prone to NETosis-primed neutrophils are attracted by intestinal fibroblast-derived IL-8, migrating in the mucosa, where they stimulate fibroblasts through the production of NET-enriched extracellular mediators (eNETs), transforming them into a KLF2 (-), CCN2 (++) phenotype that releases collagen, leading to fibrotic complications. Stimulated fibroblasts further produce IL-8, which sustains the vicious immunofibrotic cycle of neutrophil-fibroblast interaction. Pharmaceutical agents targeting IFNα or IL-8, as well as JAK-1/2 signaling in both fibroblasts and neutrophils, may be promising therapeutic candidates against Crohn’s disease. CCN2, cellular communication network factor 2; eNETs, enriched neutrophil extracellular traps; IFNα, interferon alpha; JAK, Janus kinase; KLF2, Kruppel-like factor 2. Created with Biorender.com.

Fibrosis leads to abnormal tissue remodeling complicating several chronic inflammatory diseases, while early prevention of fibrosis remains a significant unmet medical need (50). Our mechanistic studies suggest that the interplay between neutrophils/eNETs and fibroblasts in CD is an early immunofibrotic event, that may be disrupted by using various pharmaceutical agents, such as anti-IL-8 (51) to interrupt the intestinal migration of pro-fibrotic neutrophils, recombinant DNase I to dismantle the chromatin scaffold of fibrogenic eNETs (52) or inhibitors of JAK signaling (53) both in peripheral neutrophils and intestinal fibroblasts to prevent their activation (**Figure 8**). Recent experience in severe COVID-19 patients indicates that combining different immunomodulatory therapies may be beneficial for complex fibrotic diseases such as CD (13, 52). Lately, JAK inhibition has been approved as a new treatment for moderate-to-severe CD patients, further supporting the translational impact of our study (53).

Linking our findings with clinical practice, we found that levels of IFNα in serum or/and mRNA expression of selective IFN signaling-related components in peripheral neutrophils could serve as surrogate markers of CD activity, positively correlated with the CDAI, a well-established disease severity index that includes endoscopic findings (18).

In conclusion, this study unravels the role of IFNα/JAK signaling in the plasticity of neutrophils/NETs during their crosstalk with intestinal fibroblasts, eventually leading to the immunofibrosis of CD. The IFNα/neutrophil/fibroblast pathway provides novel candidate targets for the design of future diagnostic and therapeutic strategies in CD (**Figure 8**).

## Data Availability

Raw data supporting the findings of this study are available from the corresponding authors on request. The datasets presented in this study can be found in the NCBI's Sequence Read Archive (SRA) repository. The URL of the repository and accession number can be found below: https://www.ncbi.nlm.nih.gov/sra, PRJNA997815.
