# Supplementary material for: Neutrophil-fibroblast crosstalk drives immunofibrosis in Crohn’s disease through IFNα pathway
Source: Front Immunol. 2024 Sep 13;15:1447608. doi: 10.3389/fimmu.2024.1447608 (PMC11427415; doi:10.3389/fimmu.2024.1447608)
Supplement: Supplementary file 1 [file DataSheet1.pdf]

Supplementary Material

Neutrophil-fibroblast crosstalk drives immunofibrosis in Crohn's disease through IFNα pathway

Authors:

Efstratios Gavriilidis[1,2]†, Georgios Divolis[3]†, Anastasia-Maria Natsi[2]†, et al.

1 Supplementary Materials and Methods

1.1 Isolation of peripheral blood neutrophils and serum

Peripheral heparinized blood was collected and circulating neutrophils were isolated by Histopaque double-gradient density centrifugation (1119.1 g/mL and 1077.1 g/mL, Sigma-Aldrich, St Louis, MO, USA) at 700×g for 35 min at 20-25ºC. Then, cells were washed once with phosphate-buffered saline (PBS-1×, Biosera) and centrifuged at 200×g for 12 min before being cultured. Neutrophils were adjusted to the desired concentration and used within 2 h after isolation. Cell purity was estimated at ≥ 98%.

To collect serum, venous blood from either healthy individuals (HI) or patients with Crohn's disease (CD) and/or ulcerative colitis (UC) was collected in appropriate BD Vacutainer® Plus Plastic Serum tubes (Becton, Dickinson, and Company) and centrifuged at 500×g for 15 min. Serum samples were stored at -80°C until analyzed.

1.2 Isolation, culture, and characterization of human primary intestinal fibroblasts

To investigate the pro-fibrotic potential of the intestinal inflammatory environment, human primary intestinal fibroblasts (PIFs) were isolated from colonic and/or terminal ileum biopsies obtained by colonoscopy from inflammatory lesions of active UC and CD patients as described above, based on a standard lab protocol (54,55). PIFs isolated from healthy subjects who underwent preventive colonoscopy served as controls. PIFs in passages 3–6 were cultured in DMEM (31885-023; Thermo Fisher Scientific) supplemented with 10% heat-inactivated fetal bovine serum (FBS; 10082147; Thermo Fischer Scientific), 200 U/mL antibiotic/antimycotic solution (15240062; Thermo Fisher Scientific), 1% non-essential amino acids (11140-035; Thermo Fisher Scientific) (complete DMEM) at 37°C, 5% CO_2. To verify their phenotype, cells were stained using antibodies against alpha-smooth muscle actin (aSMA), vimentin, and desmin (56). PIFs were also plated in 6-well culture plates (≈0.8-1 x 10^6 cells/well; Corning Incorporated) in complete DMEM to collect their supernatant. Once cells were 60% to 70% confluent, the medium was removed, cells were rinsed in PBS, and fresh low-serum DMEM (2% FBS) was added for 24 h. PIFs' confluence was determined using optical microscopy. To avoid subjectivity errors before experimental procedures, fibroblasts' confluence was assessed by two independent experienced researchers. After 24 h, the medium was collected, and centrifuged, and the supernatant was transferred to a new tube. Aliquots of the supernatant were stored at −80°C until use.

1.3 RNA isolation, cDNA synthesis, and RT-qPCR

RNA was extracted from PIFs and peripheral blood neutrophils in TRIzol reagent following the manufacturer's protocol (15596026; Thermo Fischer Scientific). Equal amounts of RNA were used to synthesize cDNA for Real-time qPCR (RT-qPCR) analysis or to prepare cDNA libraries for RNA-Sequencing (RNA-Seq). cDNA for RT-qPCR was generated using the PrimeScript™ RT Reagent Kit (RR037A; Takara) according to the manufacturer's instructions. RT-qPCR for Krüppel-like Factor 2 (*KLF2*), Interleukin-8 (*IL8*), Signal transducer and activator of transcription 1 (*STAT1*), Signal transducer and activator of transcription 2 (*STAT2*), and/or Cellular Communication Network Factor 2 (*CCN2*) was performed using a KAPA SYBR FAST qPCR Master Mix (2X) (KK4602; KAPA Biosystems). To normalize the expression of the

abovementioned genes, glyceraldehyde 3-phosphate dehydrogenase (*GAPDH*) was used as an internal control gene. Details regarding the primer pairs and RT-qPCR conditions are given in **Supplementary Table S4**. The data were quantified and analyzed using the $2^{-\Delta\Delta Ct}$ mathematical model.

1.4 In-cell ELISA (ICE assay, Cytoblot)

To study the intracellular protein levels of CCN2 or KLF2, PIFs were cultured in 96-well high-binding microplates (655061; Thermo Fisher Scientific) in the presence of distinct stimuli for 4 h, as previously described (13,21,57). Briefly, goat anti-CCN2 (2 μg/mL; sc14939; Santa Cruz Biotechnology Inc.) or mouse anti-KLF2 (6 μg/mL; MA5-24300; Thermo Fisher Scientific) were used as primary monoclonal antibodies. Horseradish peroxidase-conjugated donkey anti-goat IgG (1:1000 dilution, HAF109; R&D Systems) or donkey anti-mouse IgG (1:1000 dilution, HAF018; R&D Systems) were used as secondary antibodies. Absorbance was measured at 620 nm. The corrections were performed by subtracting the signal of the wells incubated in the absence of the primary antibody.

1.5 Immunofluorescence staining in human PIFs

PIFs were cultured in 8-well-chambered slides (80806; Ibidi) and treated with various stimuli for 4 h, as previously described (13,52). For the assessment of collagen type I (COL1) expression, fibroblasts were treated with the specified agents for 24 h. In brief, a rabbit anti-CCN2 polyclonal antibody (1:600 dilution; ab5097; Abcam), a mouse anti-KLF2 monoclonal antibody (6 μg/mL; MA5-24300; Thermo Fisher Scientific), a mouse anti-aSMA monoclonal antibody (1:300 dilution; NBP2-34522; Novus Biologicals), a rabbit anti-Collagen type I (COL1) polyclonal antibody (1:200 dilution; NB600-408; Novus Biologicals), a mouse anti-IL-8 monoclonal antibody (15 μg/mL; MAB208-100; R&D systems), a rabbit anti-desmin polyclonal antibody (1:200 dilution; ab15200; Abcam), and/or a rabbit anti-vimentin monoclonal antibody (1:250 dilution; ab92547; Abcam), were used as primary antibodies. Afterward, a Drop-n-Stain CF488A Goat anti-mouse IgG (H+L), highly cross-absorbed antibody (20956; Biotium), or a Drop-n-Stain CF594 Goat anti-rabbit IgG (H+L), highly cross-absorbed antibody (20955; Biotium) were utilized as secondary antibodies. A mounting medium with DAPI (50011; Ibidi) was used in the final step. To distinguish specific staining from non-specific binding or background fluorescence, no primary antibody staining was used as an internal control in each experiment.

Imaging was performed on a customized Andor Revolution Spinning Disc Confocal system with a Yokogawa CSU-X1 (Yokogawa, Tokyo, Japan) confocal scan-head, built around an Olympus IX81 microscope (Olympus, Tokyo, Japan) mounted with a 40x (0.95NA) and a 20x (0.45NA) air objectives and a digital camera (Andor Zyla 4.2 sCMOS; Andor, Belfast, Ireland) (Bioimaging-DUTH facility). All system components were controlled with Andor IQ 3.6.5.

Quantification and determination of mean fluorescence intensity (MFI) from fluorescence microscopy images using confocal microscopy was performed with ImageJ software (ImageJ for Windows, Version 1.53k). The mean of three high-power fields per subject was assessed.

1.6 Collagen measurement

The soluble collagen types (I–V) were determined using a Sircol Collagen Assay Kit. Briefly, PIFs were cultured in 6-well plates (Corning Inc.), treated with various agents, and the culture supernatant was collected after 24 h, the time point at which the highest collagen production was observed, based on preliminary timepoint experiments. Cell debris was removed by centrifugation, and the resulting supernatant was incubated overnight at 4°C, with isolation and concentration reagent provided with the kit. The assay was therefore conducted according to the manufacturer's protocol (CLRS1000; Biocolor), as previously described (13,58).

1.7 Multiplex cytokine measurement

The levels of multiple inflammatory cytokines were measured in serum and PIFs supernatants using the Human Inflammation Panel 1, LEGENDplex™ Multi-Analyte Flow Assay Kit (740809; Biolegend) in a CyFlow Cube 8 flow cytometer (Sysmex Partec, Germany), according to the manufacturer's instructions as previously described (29).

1.8 Immunohistochemistry (IHC-P), Masson's trichrome, and immunofluorescence (IF) staining in tissue sections.

To evaluate the presence of neutrophils in intestinal tissue biopsies, cross-sections of 4 μm thickness were stained using a mouse anti-human neutrophil elastase monoclonal antibody (NE; 1:50 dilution; clone NP57; DAKO). Serial cross-sections obtained from the same intestinal biopsies were further stained with Masson's trichrome to identify the presence of intestinal fibrosis, following the manufacturer's protocol (Masson-Goldner Trichrome kit, MGT-100T; Biognost). Samples were visualized under light microscopy (Nikon, model Eclipse E400) and images were captured using a Nikon Digital Camera (ACT-1 Nikon software).

Immunofluorescence staining was further performed in formalin-fixed paraffin-embedded biopsies, as previously described (59,60). Briefly, nonspecific binding sites were blocked with 2% normal goat serum in 2% BSA-PBS. Paraffin-embedded tissue sections were deparaffinized and stained with a rabbit anti-aSMA monoclonal antibody (1:100 dilution; 701457; Invitrogen), a rabbit anti-vimentin monoclonal antibody (1:650 dilution; ab92547; Abcam), a mouse anti-KLF2 monoclonal antibody (6 μg/mL; MA5-24300; Thermo Fisher Scientific), a mouse anti-IL-8 monoclonal antibody (15 μg/mL; MAB208-100; R&D systems), a mouse anti-aSMA monoclonal antibody (1:300 dilution; NBP2-34522; Novus Biologicals), and a rabbit anti-COL1 polyclonal antibody (1:200 dilution; NB600-408; Novus Biologicals). The next day and after extensive washes in PBS-1×, a Drop-n-Stain CF488A Goat anti-mouse IgG (H+L), highly cross-absorbed antibody (20956; Biotium), or a Drop-n-Stain CF594 Goat anti-rabbit IgG (H+L), highly cross-absorbed antibody (20955; Biotium) were utilized as the secondary antibodies. A rabbit IgG polyclonal antibody (isotype control; ab171870; Abcam) or a mouse IgG polyclonal antibody (isotype control; ab37355; Abcam) were used as controls. DNA was counterstained by DAPI (D9542; Sigma-Aldrich), and samples were mounted for visualization. Imaging was performed on a customized Andor Revolution Spinning Disc Confocal system with a Yokogawa CSU-X1 (Yokogawa, Tokyo, Japan) confocal scan-head, built around an Olympus IX81 microscope (Olympus, Tokyo, Japan) mounted with a 40x (0.95NA) and a 20x (0.45NA) air objectives and a digital camera (Andor Zyla 4.2 sCMOS; Andor, Belfast, Ireland) (Bioimaging-DUTH facility). All system components were controlled with Andor IQ 3.6.5.

1.9 *In-vitro* transwell migration assay (chemotaxis assay)

To evaluate neutrophil chemotaxis, low-serum complete DMEM suitable for the growth of intestinal fibroblasts (served as blank) and/or supernatant from HI, UC, and CD PIFs were added to the lower chamber of a 24-well QCM^{TM} chemotaxis 3 µm cell migration assay (ECM505; Merck Millipore), as described in the manufacturer's instructions (61). In brief, freshly isolated human peripheral blood neutrophils (0.2-2 x 10^6 cells/mL) were seeded in the upper well in low-serum complete DMEM. After 2.5 h of incubation at 37ºC and 5% CO_2, migrating cells were quantified by measuring fluorescence using a Perkin Elmer fluorescence plate reader (Enspire, Waltham, MA, USA), set at excitation/emission wavelengths of 480/520 nm. Four independent experiments were performed, using neutrophils from a different healthy donor each time, in which mean fluorescence intensity was evaluated. As an inhibitor of neutrophil chemotaxis, a mouse anti-IL-8/CXCL8 monoclonal antibody with neutralizing effect (0.3 µg/mL; MAB208-100; R&D systems) was used, while an IgG rabbit polyclonal antibody (0.3 µg/mL; ab171870; Abcam) was used as a control in neutralization experiments.

2 Supplementary Figures

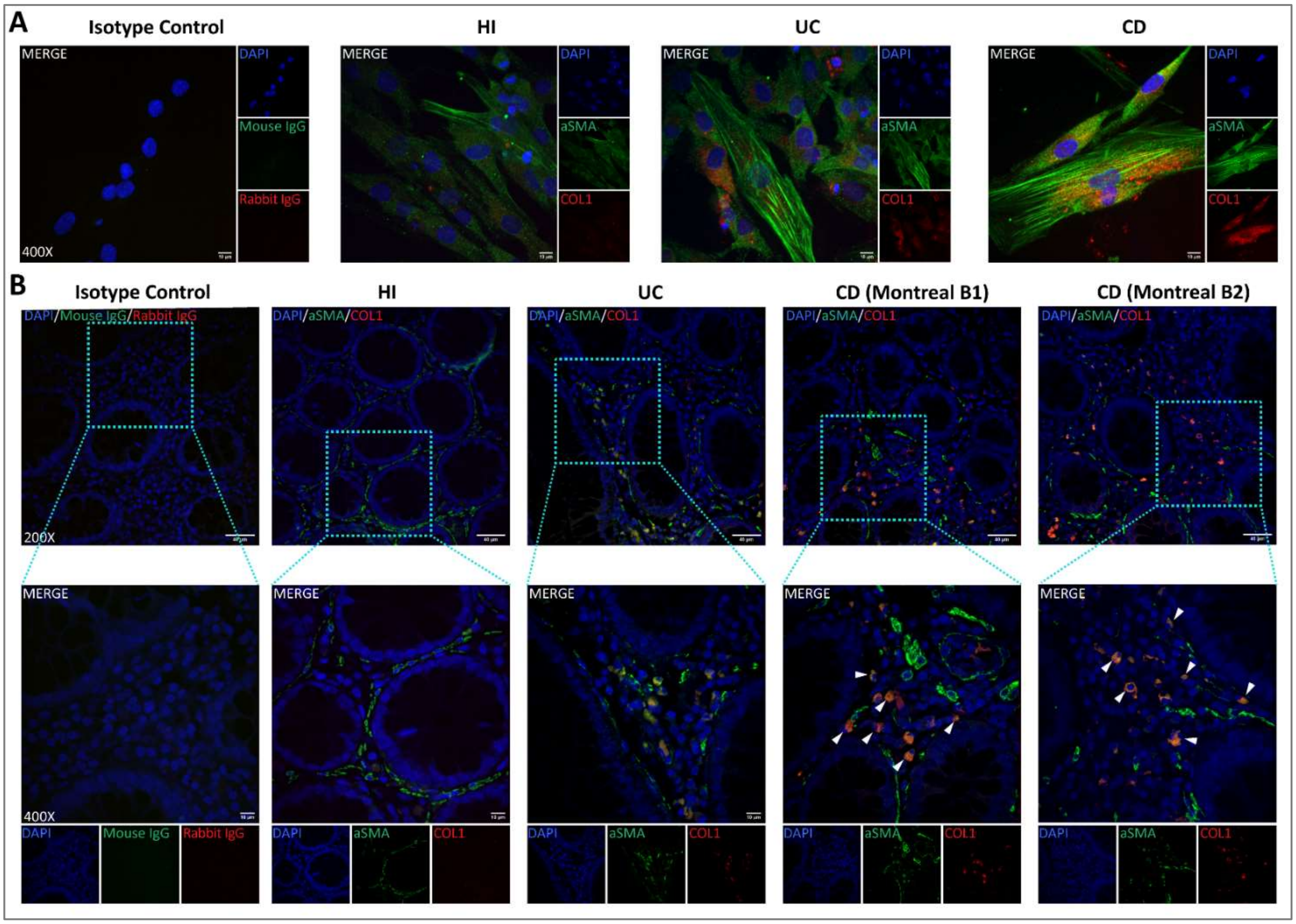

Supplementary Figure S1. CD intestinal tissue fibroblasts are characterized by increased aSMA and collagen type I immunoreactivity. (A) Immunostaining (blue: DAPI, green: aSMA, red: COL1) in PIFs isolated from HI, UC, and CD patients. **(B)** Immunostaining (blue: DAPI, green: aSMA, red: COL1) in tissue sections obtained from HI, UC, CD patients with inflammatory, non-stricturing disease (Montreal B1), and CD patients with inflammatory strictures (Montreal B2). Dotted frames represent the zoomed-in areas shown below. White arrowheads in **(B)** show double-positive aSMA/COL1 cells observed in CD patients. **(A)**, **(B)** Isotype controls are provided. One representative example is shown. Confocal Microscopy. **(A)**, **(B)** Magnification: 200x, scale bar: 40μm; framed areas in panel **(B)**: Magnification: 400x, scale bar: 10μm. aSMA, alpha-smooth muscle actin; CD, Crohn's disease; COL1, collagen type I; HI, healthy individuals; UC, ulcerative colitis.

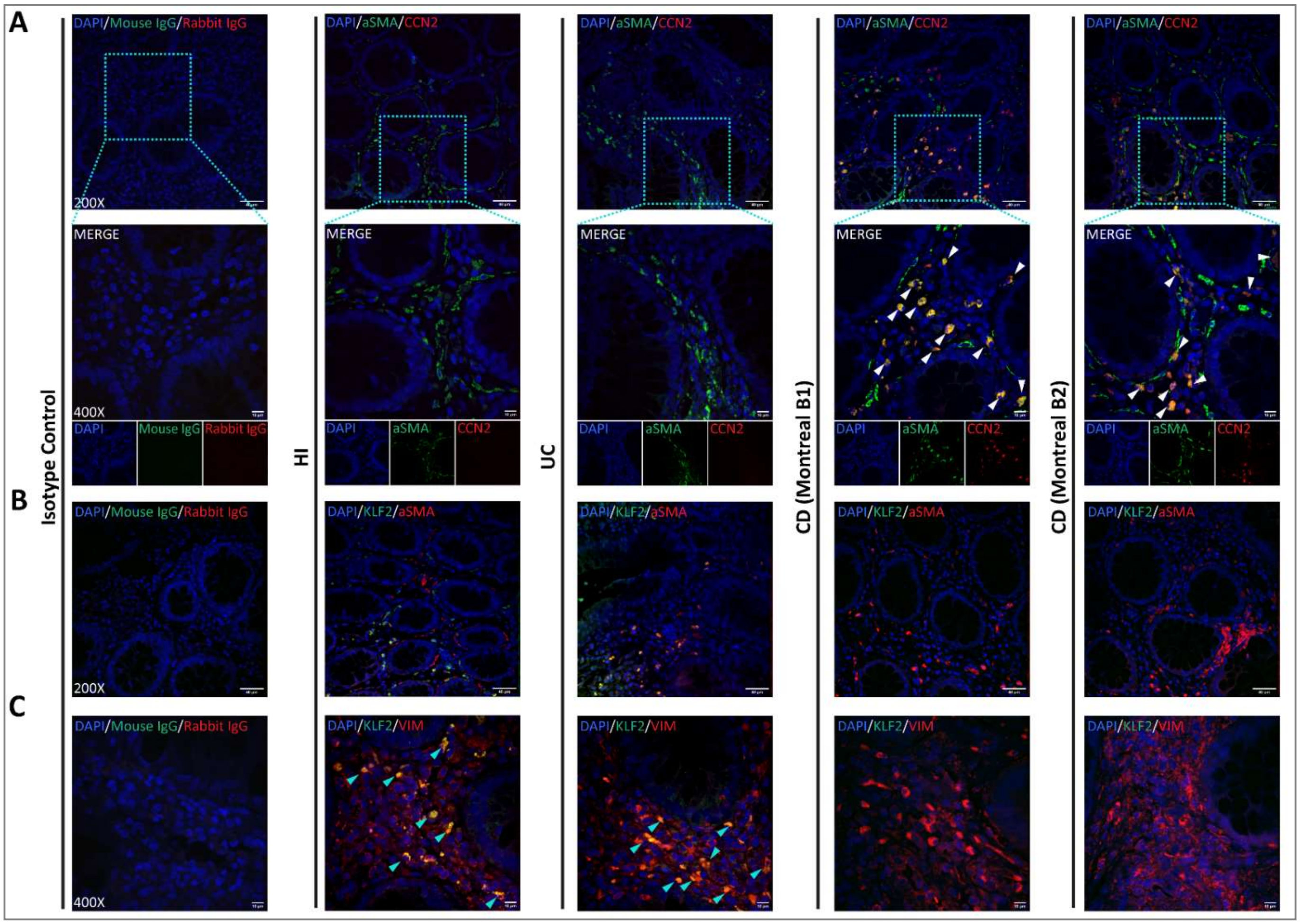

Supplementary Figure S2. CD intestinal tissue fibroblasts exhibit enhanced CCN2 and reduced KLF2 immunoreactivity. Immunostainings **(A)** blue: DAPI, green: aSMA, red: CCN2, **(B)** blue: DAPI, green: KLF2, red: aSMA, **(C)** blue: DAPI, green: KLF2, red: vimentin, in tissue sections obtained from HI, UC, and CD patients either with inflammatory, non-stricturing disease (Montreal B1) or with inflammatory strictures (Montreal B2). Dotted frames in **(A)** represent the zoomed-in areas shown below. White arrowheads in **(A)** show double-positive aSMA/CCN2 cells observed in CD patients. Cyan arrows in **(C)** show double-positive KLF2/vimentin cells observed in HI and UC patients. **(A-C)** Isotype controls are provided; one representative example is shown. **(A)** Magnification: 200x, scale bar: 40μm; Framed areas in panel **(A)** Magnification: 400x, scale bar: 10μm. **(B)** Magnification: 200x, scale bar: 40μm. **(C)** Magnification: 400x, scale bar: 10μm. aSMA, alpha-smooth muscle actin; CD, Crohn's disease; CCN2, cellular communication network factor 2; KLF2, Kruppel-like factor 2; HI, healthy individuals; UC, ulcerative colitis; VIM, vimentin.

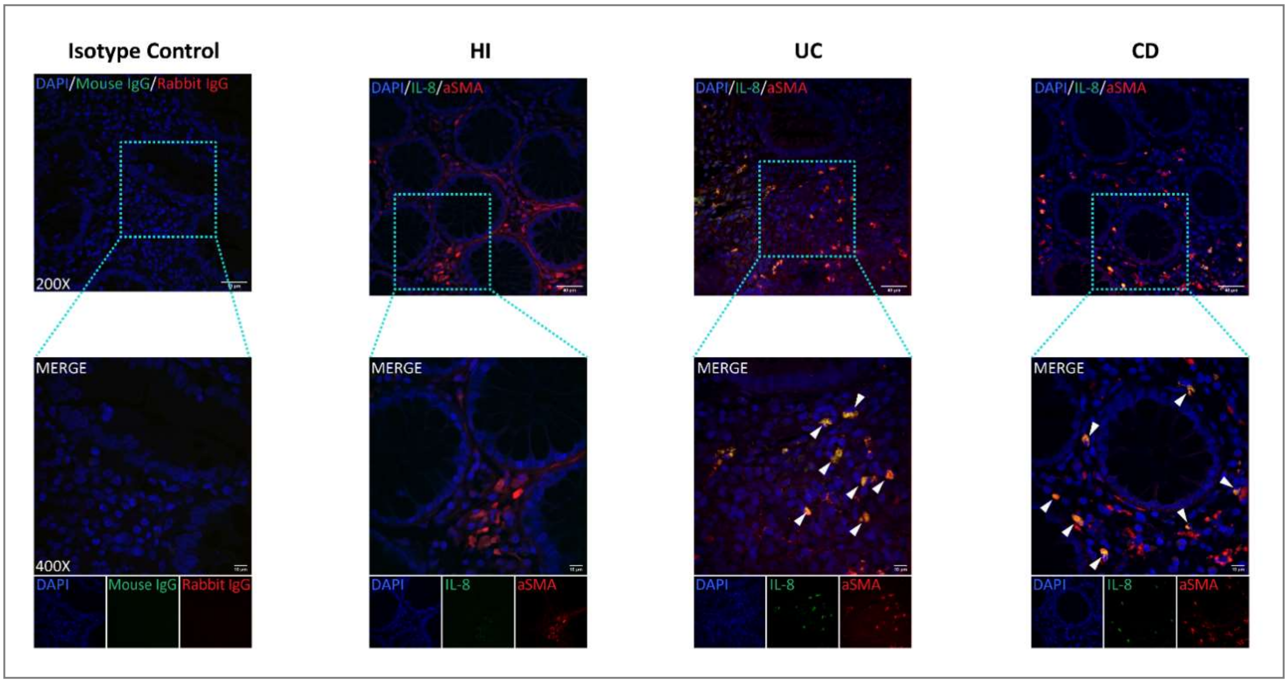

Supplementary Figure S3. UC and CD myofibroblasts are positive for IL-8 immunostaining. Immunostaining (blue: DAPI, green: IL-8, red: aSMA) in tissue sections obtained from HI, UC, and CD patients. Magnification: 200x, scale bar: 40μm. Dotted frames represent the zoomed-in areas shown below (magnification: 400x, scale bar: 10μm). Isotype controls are provided; one representative example is shown. White arrowheads show IL-8/aSMA-positive cells observed in UC and CD patients. aSMA, alpha-smooth muscle actin; CD, Crohn's disease; HI, healthy individuals; UC, ulcerative colitis.

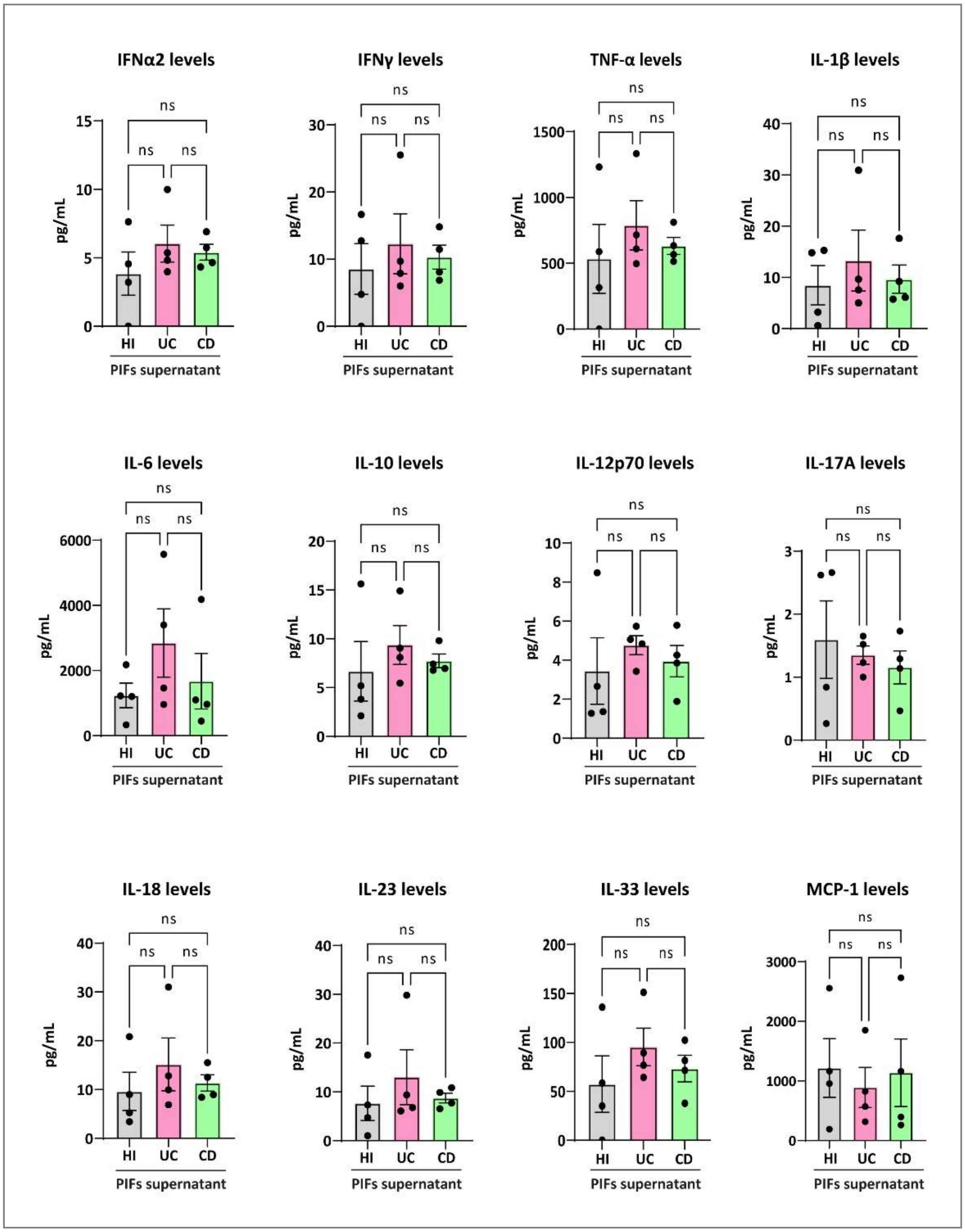

Supplementary Figure S4. Inflammatory cytokines in the supernatants of PIFs obtained from IBD patients and HI. Concentration of IFNα2, IFNγ, TNF-α, IL-1β, IL-6, IL-10, IL-12p70, IL-17A, IL-18, IL-23, IL-33, and MCP-1 in the supernatants of *ex-vivo* isolated PIFs (*n=4/group*), assessed by a bead-based multiplex flow cytometric assay. Nonparametric Kruskal-Wallis followed by Dunn's multiple comparisons test was performed, ns: not significant. Data are expressed as mean ± SEM. CD, Crohn's disease; HI, healthy individuals; MCP-1, monocyte chemoattractant protein-1; PIFs, primary intestinal fibroblasts; TNF-α, tumor necrosis factor alpha; UC, ulcerative colitis.

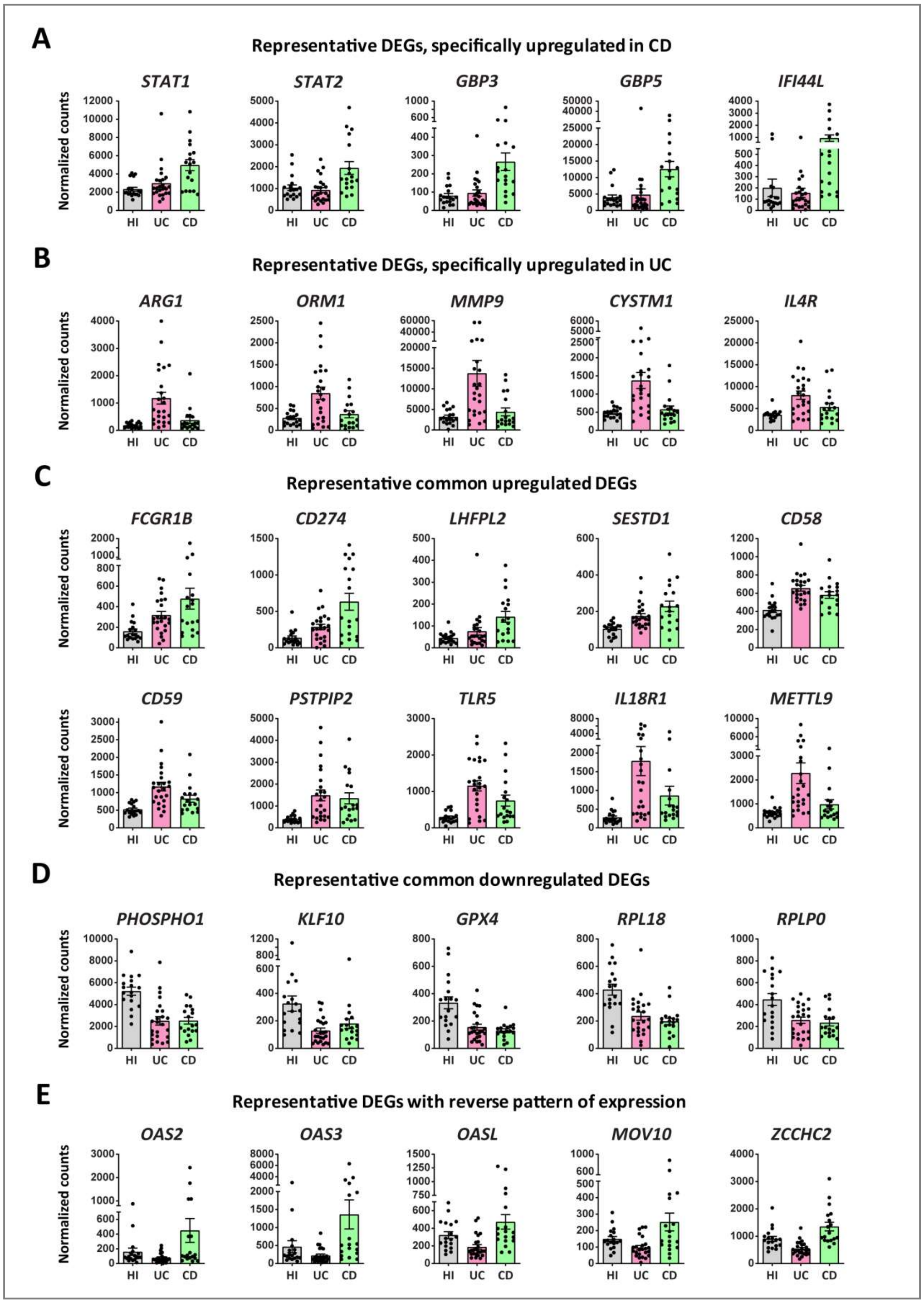

Supplementary Figure S5. Representative differentially expressed genes in peripheral blood neutrophils isolated from patients with IBD and HI. Graphs of representative DEGs (baseMean > 30 and FDR < 0.05), specifically upregulated in CD **(A)** or UC **(B)**, commonly upregulated **(C)** or downregulated **(D)**, or showing a reverse expression pattern **(E)**, following RNA-Seq analysis of peripheral blood neutrophils from patients with CD (*n=18*) or UC (*n=24*). DEGs were identified following comparison with neutrophils isolated from HI (*n=18*). CD, Crohn's disease; DEGs, differentially expressed genes; HI, healthy individuals; UC, ulcerative colitis.

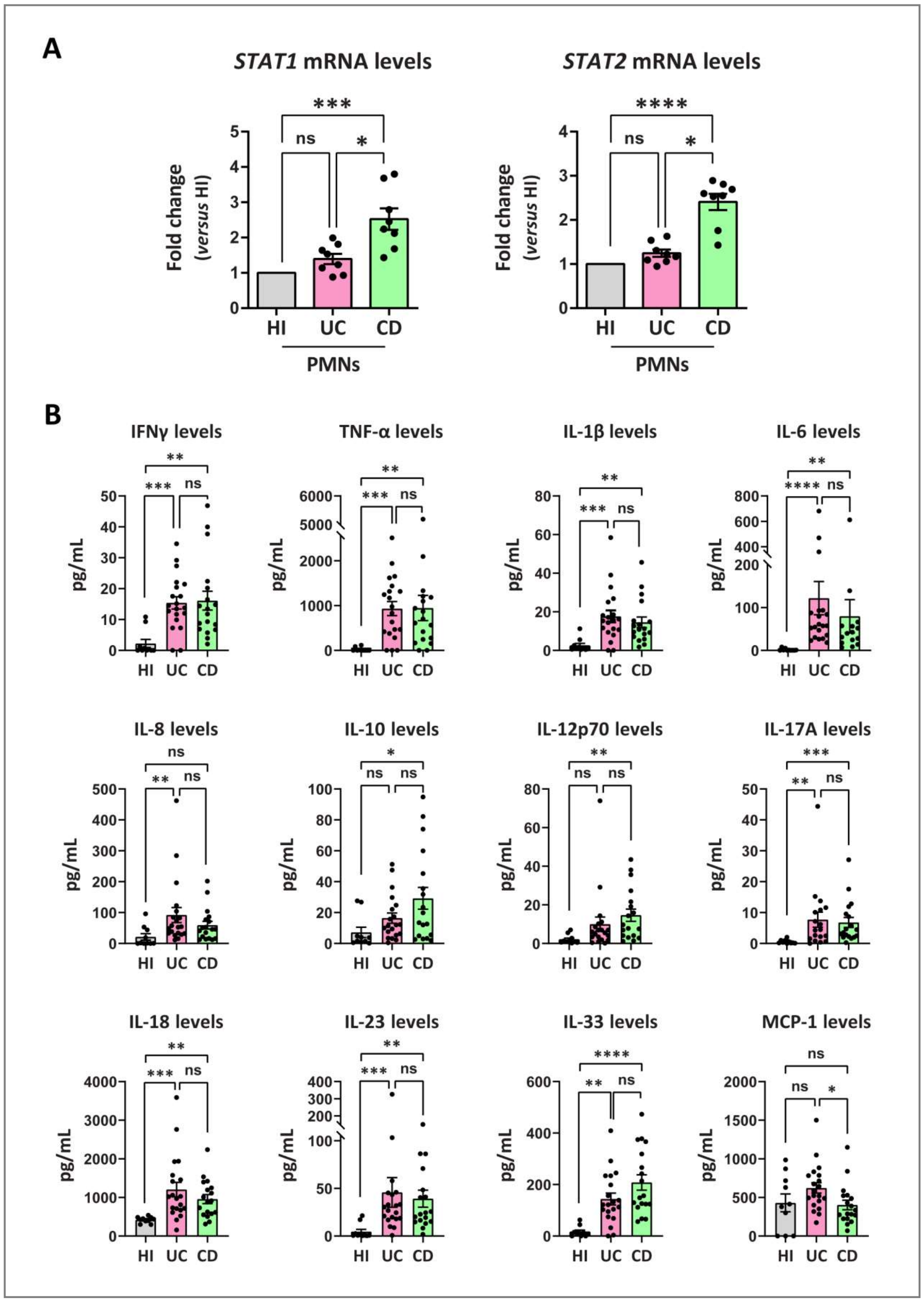

Supplementary Figure S6. ***STAT1*** **and** ***STAT2*** **mRNA levels in peripheral neutrophils and cytokine analysis in serum. (A)** *STAT1* and *STAT2* mRNA levels of CD and UC patients, and HI, assessed by RT-qPCR (*n=8*). **(B)** Serum levels of IFNγ, TNF-α, IL-1β, IL-6, IL-8, IL-10, IL-12p70, IL-17A, IL-18, IL-23, IL-33, and MCP-1, assessed by a bead-based multiplex flow cytometric assay in HI (*n=10*), CD (*n=18*), and UC (*n=20*) patients. **(A, B)** Nonparametric Kruskal-Wallis followed by Dunn's multiple comparisons test was performed, *p<0.05, **p<0.01, ***p<0.001, ****p<0.0001, ns: not significant. Data are expressed as mean ± SEM. CD, Crohn's disease; HI, healthy individuals; IFN, interferon; MCP-1, monocyte chemoattractant protein-1; STAT, signal transducer and activator of transcription; TNF-α, tumor necrosis factor alpha; UC, ulcerative colitis.

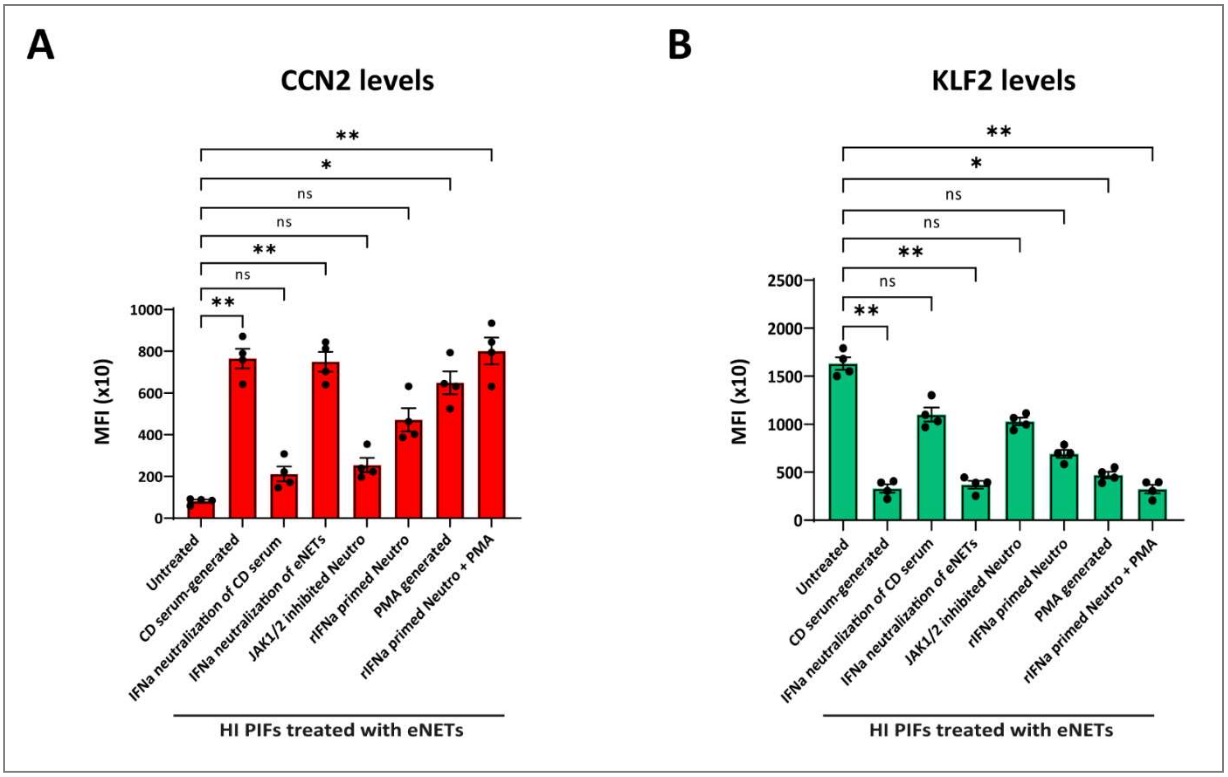

Supplementary Figure S7. MFI quantification of CCN2 and KLF2 immunoreactivity in HI PIFs treated with *in-vitro* generated eNETs. (A) Quantification of CCN2 and **(B)** KLF2 levels. Nonparametric Kruskal-Wallis followed by Dunn's multiple comparisons test was performed, *n=4/group*, *p<0.05, **p<0.01, ns: not significant. Data are expressed as mean ± SEM. CCN2, cellular communication network factor 2; eNETs, enriched neutrophil extracellular traps; KLF2, Kruppel-like factor 2; MFI, mean fluorescence intensity; PMA, phorbol-12-myristate-13-acetate.

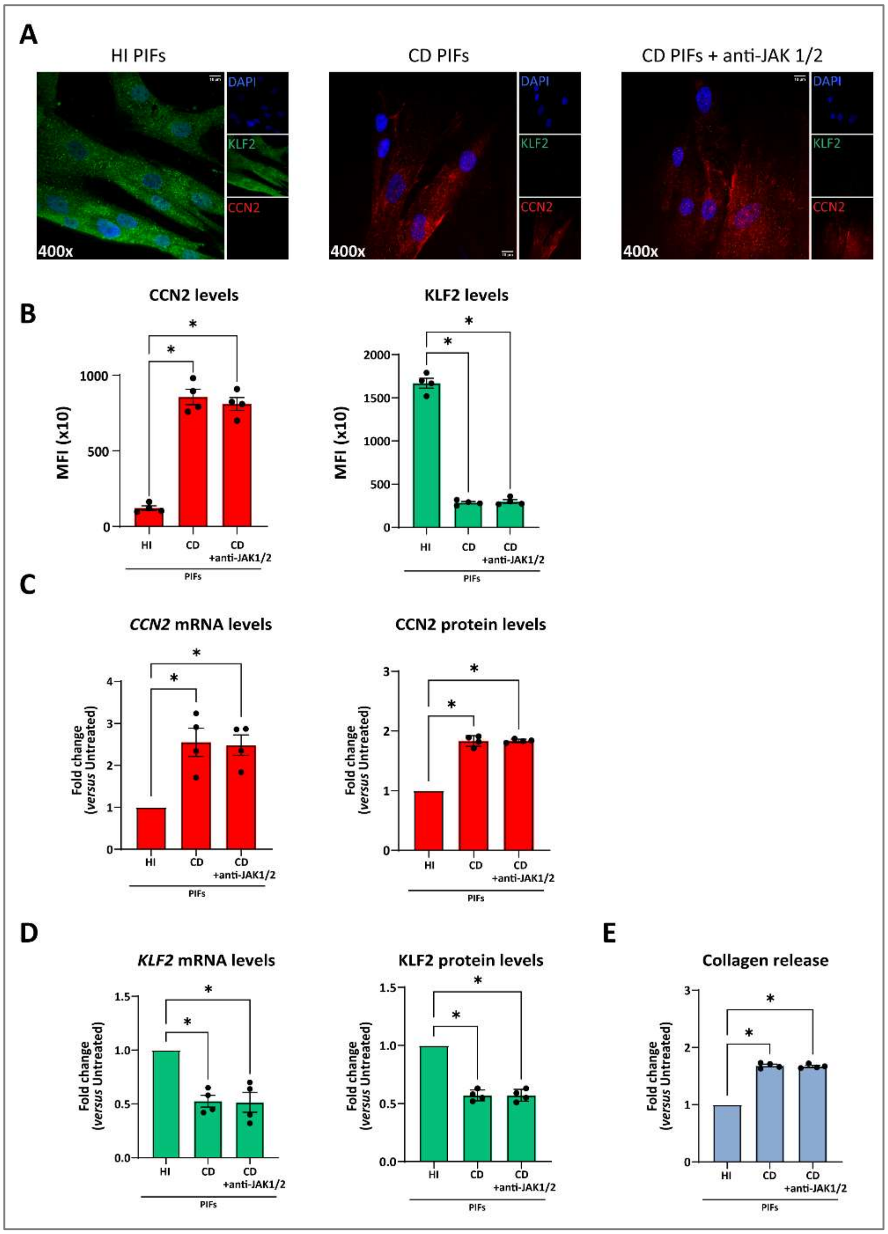

Supplementary Figure S8. The fibrotic phenotype of CD PIFs is not reversed to normal by direct JAK-1/2 inhibition. JAK-1/2 inhibitor, baricitinib, was used to treat *ex-vivo* isolated CD PIFs. Untreated CD and HI PIFs were used as comparators. **(A)** Immunofluorescence showing KLF2 and CCN2 levels (blue: DAPI, green: KLF2, red: CCN2) and **(B)** corresponding MFI quantification. **(C)** CCN2 and **(D)** KLF2 mRNA and protein levels as assessed by RT-qPCR and in-cell ELISA, respectively. **(E)** Collagen release assay results following the above setups. **(A)** One representative example out of four independent experiments is shown. Confocal microscopy. Magnification: 400x, Scale Bar: 10μm. **(B-E)** Nonparametric Kruskal-Wallis followed by Dunn's multiple comparisons test was performed, *n=4/group*, *p<0.05. Data are expressed as mean ± SEM. CCN2, cellular communication network factor 2; CD, Crohn's disease; JAK, Janus kinase; KLF2, Kruppel-like factor 2; MFI, mean fluorescence intensity; PIFs, primary intestinal fibroblasts.

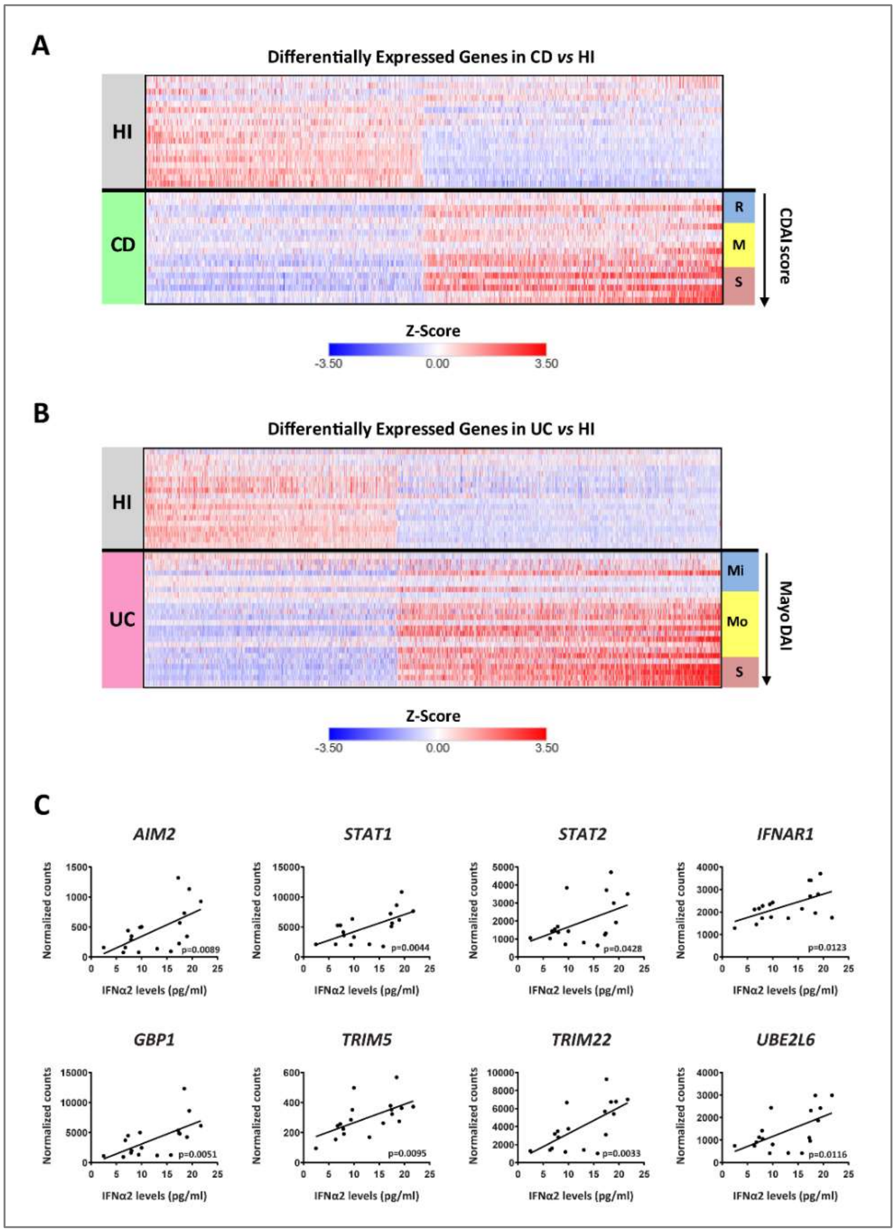

Supplementary Figure S9. Transcriptomic alterations in peripheral blood neutrophils correlate with disease severity in IBD patients. (A, B) Heatmaps depicting the relative expression of all DEGs (baseMean > 30 and FDR < 0.05), as determined by RNA-Seq analysis of neutrophils isolated from HI (*n=18*) and CD (*n=18*, **A**) or UC patients (*n=24*, **B**). CDAI and Mayo DAI were used for assessing disease activity in CD and UC, respectively. **(C)** Correlation plots of the mRNA expression of key interferon signaling components, as determined by RNA-Seq analysis of CD neutrophils, *versus* serum IFNα2 levels. Simple linear regression was used to assess the relationship between normalized counts and IFNα2 levels. CD, Crohn's disease; CDAI, Crohn's disease activity index (R, remission, < 150; M, mild to moderate, 150-220; S, moderate to severe, > 220); FDR, false discovery rate; HI, healthy individuals; IBD, inflammatory bowel disease; Mayo DAI, Mayo score disease activity index (Mi, mild, 3-5; Mo, moderate, 6-10; S, severe, 11-12); UC, ulcerative colitis.

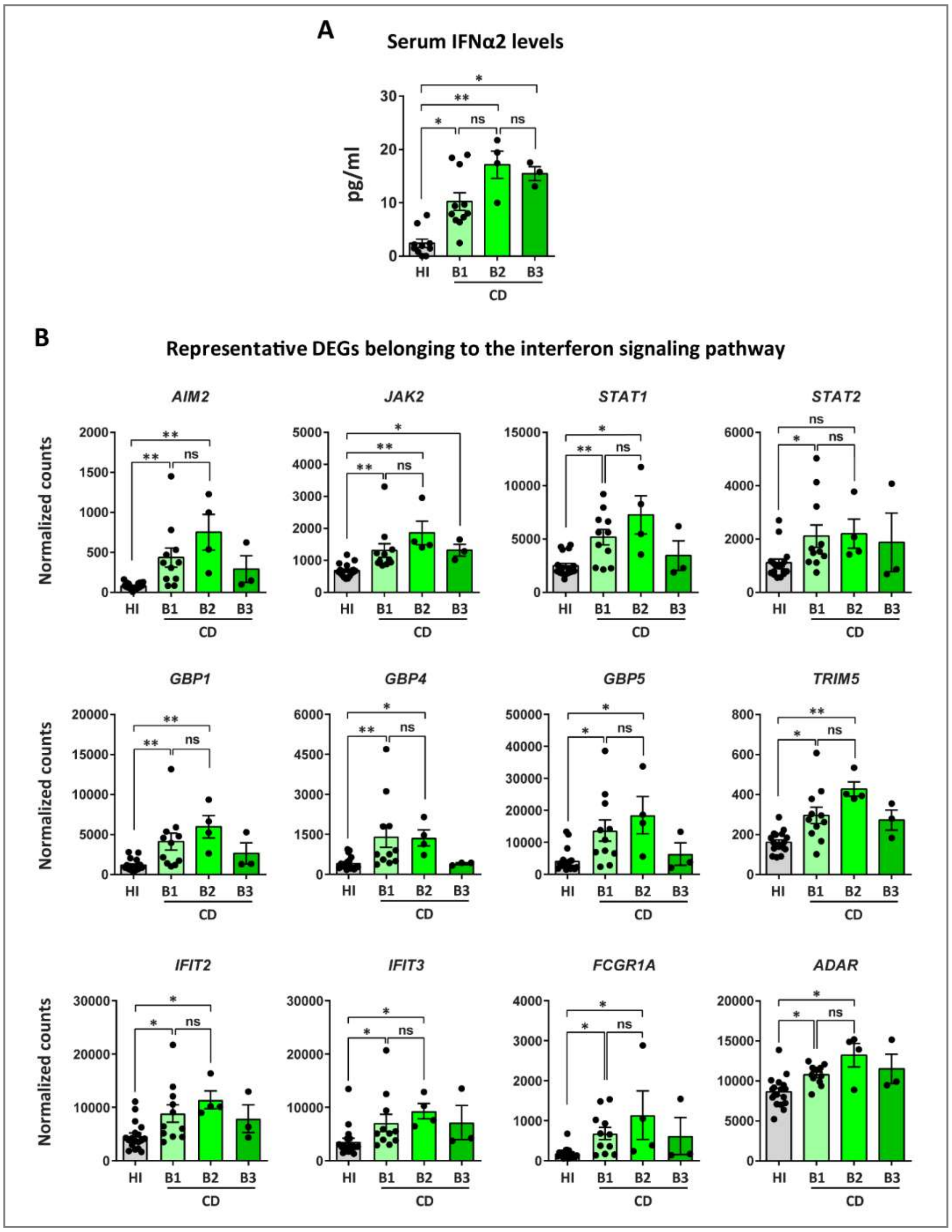

Supplementary Figure S10. Concentration of IFNα2 in the serum and expression of IFN signaling-related genes in peripheral neutrophils from HI and CD patients characterized by B1, B2, or B3 disease states, according to the Montreal score. (A) Levels of IFNα2 in the serum of HI and patients with CD, as assessed by a bead-based multiplex flow cytometric assay. **(B)** Graphs of representative DEGs (baseMean > 30 and FDR < 0.05), belonging to the interferon signaling pathway, following RNA-Seq analysis of peripheral blood neutrophils from patients with CD (*n=18*). DEGs were identified following comparison with neutrophils isolated from HI (*n=18*). Data are expressed as Mean ± SEM. Nonparametric Kruskal-Wallis test was applied, followed by Dunn's multiple comparisons test, *p<0.05, **p<0.01, ns: not significant. Montreal score according to disease behavior: B1, non-stricturing, non-penetrating (*n=11*); B2, stricturing (inflammatory strictures, *n=4*); B3, penetrating (*n=3*); CD, Crohn's disease; DEGs, differentially expressed genes; HI, healthy individuals.

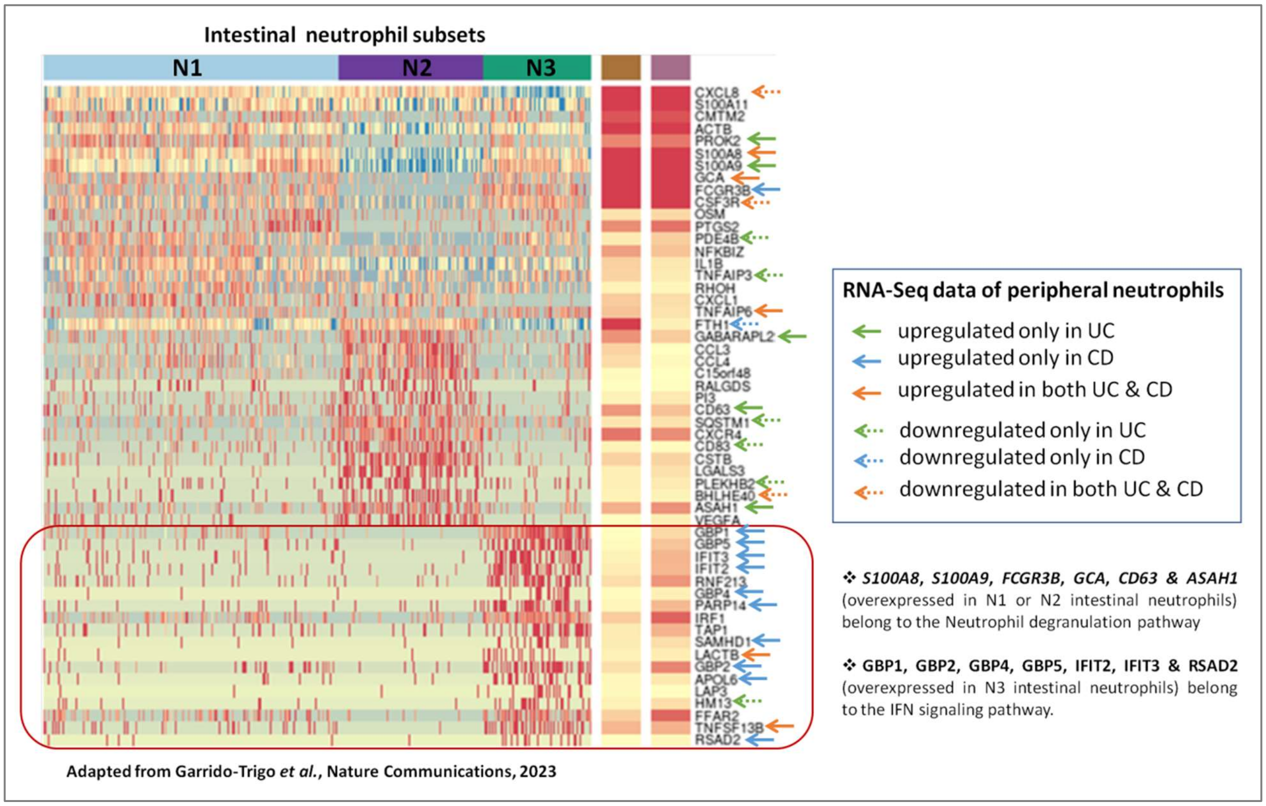

Supplementary Figure S11. Comparison of transcriptomic data between intestinal (Garrido-Trigo *et al*., 2023) and peripheral blood neutrophils (current manuscript) of IBD patients. Heatmap showing the average normalized and scaled expression of differentially expressed genes in three intestinal neutrophil subsets (N1, N2, and N3) identified by single-cell analysis of intestinal tissues isolated from IBD patients, adapted by Garrido-Trigo *et al*., Nature Communications, 2023, licensed under the Creative Commons Attribution 4.0 International License. The colored arrows depict common regulated genes between these intestinal neutrophils and the peripheral neutrophils presented in the current manuscript. Peripheral blood neutrophils were isolated from patients with CD (*n=18*) or UC (*n=24*). Differentially expressed genes (baseMean > 30 and FDR < 0.05) were identified following comparison with neutrophils isolated from healthy individuals (*n=18*). Peripheral UC neutrophils differentially express genes identified in the N1 and N2 intestinal populations, including transcripts related to the neutrophil degranulation pathway, whereas peripheral CD neutrophils overexpress genes related to the N3 signature of intestinal neutrophils (12 out of 18 genes identified in N3 neutrophils), sharing a marked IFN-response signature. CD, Crohn's disease; IBD, inflammatory bowel disease; UC, ulcerative colitis.

3 Supplementary Tables

Table S1. Characteristics of healthy individuals and samples used (✓) in experiments.

Sample	Sex/Age (y)	RNA-Seq	Serum	PIFs	eNETs	Tissue	RT-qPCR
HI1	M/24	✓					
HI2	F/25	✓					
HI3	F/30	✓	✓		✓		✓
HI4	M/62	✓					
HI5	M/58	✓					
HI6	M/47	✓	✓				✓
HI7	F/23	✓	✓				
HI8	M/24	✓	✓				
HI9	M/39	✓	✓		✓		✓
HI10	F/34	✓					
HI11	F/57	✓					
HI12	M/45	✓	✓	✓	✓	✓	✓
HI13	M/55	✓	✓	✓	✓	✓	✓
HI14	M/34	✓	✓	✓	✓	✓	✓
HI15	M/32	✓					
HI16	F/38	✓	✓	✓	✓	✓	✓
HI17	M/35	✓					
HI18	M/42	✓	✓				✓

Table S2. Characteristics of CD patients and samples used (✓) in experiments.

Sample	Sex/ Age (y)	†Montreal score	#CDAI	Treatment at sampling	RNA-Seq	Serum	PIFs	eNETs	Tissue	RT-qPCR
CD1	M/29	A2,L3,B1	165	Naive	✓	✓				
CD2	M/29	A2,L1,B1	158	Naive	✓	✓				
CD3	M/19	A2,L1,B2	275	Naive	✓	✓	✓	✓	✓	
CD4	F/45	A3,L1,B1	104	Naive	✓	✓				
CD5	F/50	A3,L2,B1	89	Naive	✓	✓				
CD6	M/43	A3,L3,B1	232	Naive	✓	✓		✓		
CD7	M/17	A2,L3,B3p	295	Naive	✓	✓	✓	✓	✓	
CD8	F/50	A3,L3,B1	176	Naive	✓	✓				
CD9	F/33	A2,L1,B1	122	Naive	✓	✓				
CD10	F/39	A2,L1,B1	232	Naive	✓	✓	✓	✓	✓	
CD11	F/24	A2,L1,B1	136	Naive	✓	✓				
CD12	M/27	A2,L3,B3	160	Naive	✓	✓				
CD13	M/43	A2,L3,B3p	161	Naive	✓	✓				
CD14	M/17	A2,L1,B2	182	Naive	✓	✓				
CD15	F/65	A3,L1,B1	251	Naive	✓	✓		✓		
CD16	M/22	A2,L1,B2	274	Naive	✓	✓	✓	✓	✓	
CD17	M/39	A2,L1,B2	190	Naive	✓	✓				
CD18	M/40	A2,L2,B1	132	Naive	✓	✓				
CD19	M/32	A2,L2,B3	288	Naive						✓
CD20	M/17	A1,L1,B1	244	Naive						✓
CD21	F/20	A2,L1,B2	210	Naive						✓
CD22	M/43	A3,L2,B1	207	Naive						✓
CD23	F/34	A2,L1,B1	190	Naive						✓
CD24	M/76	A3,L1,B1	210	Naive						✓
CD25	M/45	A3,L2,B3	272	Naive						✓
CD26	F/20	A2,L2,B1	235	Naive						✓

†Montreal score classifies Crohn's disease according to (A) age of disease onset, (L) location of disease, and (B) disease behavior. A1: below 16 years; A2: between 17 and 40 years; A3: above 40 years. L1: terminal ileum; L2: colon; L3: ileocolonic. B1: non-stricturing, non-penetrating; B2: stricturing (inflammatory strictures); B3: penetrating; "p": perianal disease modifier.
#CDAI, Crohn's disease activity index. 0-150 points: disease remission; 150-220 points: mildly to moderately active disease; above 220 points: moderately to severely active disease.

Table S3. Characteristics of UC patients and samples used (✓) in experiments.

Sample	Sex/ Age (y)	*Montreal Score	$Mayo - DAI	Treatment at sampling	RNA-Seq	Serum	PIFs	eNETs	Tissue	RT-qPCR
UC1	F/25	E3,S2	9	Naive	✓					
UC2	M/66	E2,S2	4	Naive	✓	✓				
UC3	M/65	E3,S3	11	Naive	✓	✓		✓		
UC4	M/30	E2,S2	6	Naive	✓					
UC5	M/56	E2,S1	5	Naive	✓	✓				
UC6	M/42	E3,S2	8	Naive	✓	✓				
UC7	M/55	E2,S2	6	Naive	✓	✓				
UC8	M/15	E1,S1	3	Naive	✓	✓				
UC9	M/62	E3,S1	5	Naive	✓	✓				
UC10	M/47	E1,S2	7	5-ASA^	✓					
UC11	M/42	E3,S1	6	5-ASA	✓	✓				
UC12	M/70	E1,S1	4	Naive	✓	✓				
UC13	F/22	E2,S2	6	Naive	✓	✓				
UC14	M/74	E3,S3	12	Naive	✓	✓	✓	✓	✓	
UC15	M/50	E2,S3	11	Naive	✓	✓				
UC16	M/64	E3,S2	9	Naive	✓	✓	✓	✓	✓	
UC17	M/67	E3,S3	12	Naive	✓	✓	✓	✓	✓	
UC18	M/67	E3,S1	4	5-ASA	✓	✓				
UC19	F/70	E2,S1	5	5-ASA	✓					
UC20	M/66	E3,S2	8	Naive	✓	✓		✓		
UC21	F/43	E3,S3	11	Naive	✓	✓	✓	✓	✓	
UC22	M/58	E3,S2	9	5-ASA	✓	✓				
UC23	F/82	E3,S2	7	5-ASA	✓	✓				
UC24	M/37	E2,S2	6	5-ASA	✓	✓				
UC25	F/41	E3,S3	11	Naive						✓
UC26	F/78	E3,S3	11	Naive						✓
UC27	M/33	E3,S3	11	Naive						✓
UC28	F/66	E2,S2	9	Naive						✓
UC29	M/18	E2,S2	7	Naive						✓
UC30	M/32	E2,S2	6	Naive						✓
UC31	M/46	E1,S2	6	Naive						✓
UC32	M/83	E3,S2	9	Naive						✓

*Montreal score classifies ulcerative colitis according to extend (E) and severity (S). E1: ulcerative proctitis; E2: left-sided colitis; E3: extensive ulcerative colitis (pancolitis). S0: remission, no symptoms of UC; S1: mild UC, ≤ 4 bloody stools daily, lack of fever, pulse < 90 bpm, hemoglobin > 105 g/L, ESR < 30 mm/h; S2: moderate ulcerative colitis: > 4-5 stools daily but with minimal signs of systemic toxicity; S3: severe ulcerative colitis, ≥ 6 bloody stools daily, pulse > 90 bpm, temperatures > 37.5°C, hemoglobin < 10.5 g/dL, ESR > 30 mm/h.

$Mayo-DAI, Mayo disease activity index. 0-2 points: disease remission; 3-5 points: mildly active disease; 6-10 points: moderately active disease; 11-12 points: severely active disease.

^5-ASA, 5-aminosalycylic acid; 5-ASA was discontinued at least 48 hours before sampling.

Table S4. Primer sequences and RT-qPCR conditions.

Gene	Primer[1]	Primer sequence	RT-qPCR conditions[2]
$GAPDH_1$	Forward:	5′ GGGAAGCTTGTCATCAATGG 3′	**1.** 52°C for 5 min<br>**2.** 95°C for 2 min<br>**3.** 35 cycles of:<br>➢ 95°C for 15 sec<br>➢ 52°C for 40 sec<br>**4.** 52°C for 5 min<br>**5.** Melting curve analysis
	Reverse:	5' CATCGCCCCACTTGATTTTG 3′	
KLF2	Forward:	5′ AGTTCGCATCTGAAGGC 3′	
	Reverse:	5′ TGTGCTTTCGGTAGTGG 3′	
$GAPDH_2$	Forward:	5′ AGGTGGTCTCCTCTGACTTC 3′	**1.** 52°C for 5 min<br>**2.** 95°C for 2 min<br>**3.** 35 cycles of:<br>➢ 95°C for 15 sec<br>➢ 56°C for 40 sec<br>**4.** 52°C for 5 min<br>**5.** Melting curve analysis
	Reverse:	5′ CTGTTGCTGTAGCCAAATTCG 3′	
CCN2 (CTGF)	Forward:	5′ ACCAATGACAACGCCTCCTG 3′	
	Reverse:	5′ TTGCCCTTCTTAATGTTCTCTTCC 3′	
IL-8 (CXCL8)	Forward:	5’ TCTGTGTGAAGGTGCAGTTTTG 3’	
	Reverse:	5’ TGGTCCACTCTCAATCACTCTC 3’	
STAT1	Forward:	5’ CCTTGCAGAACAGAGAACACG 3’	**1.** 52°C for 5 min<br>**2.** 95°C for 2 min<br>**3.** 35 cycles of:<br>➢ 95°C for 15 sec<br>➢ 56°C for 20 sec<br>➢ 70°C for 5 sec<br>**4.** 52°C for 5 min<br>**5.** Melting curve analysis
	Reverse:	5’ GGCATTCTGGGTAAGTTCAGTG 3’	
STAT2	Forward:	5’ ACCAGAACTGGCAGGAAGC 3’	
	Reverse:	5’ AATGTCCCGGCAGAATTTCC 3’	

[1]Oligonucleotide primers were designed using Beacon Designer™ ver. 4.0.

[2]Real-time qPCR was performed using SYBR® Green RT-qPCR master mix (2x) (KAPA Biosystems) on Eco 48 Real-time qPCR (PCRmax Eco 48).

4 Supplementary References

54. Kouroumalis A, Nibbs RJ, Aptel H, Wright KL, Kolios G, Ward SG. The chemokines CXCL9, CXCL10, and CXCL11 differentially stimulate G alpha i-independent signaling and actin responses in human intestinal myofibroblasts. *J Immunol* (2005) 175:5403–5411. doi: 10.4049/jimmunol.175.8.5403
55. Kambas K, Chrysanthopoulou A, Kourtzelis I, Skordala M, Mitroulis I, Rafail S, Vradelis S, Sigalas I, Wu Y-Q, Speletas M, et al. Endothelin-1 signaling promotes fibrosis in vitro in a bronchopulmonary dysplasia model by activating the extrinsic coagulation cascade. *J Immunol* (2011) 186:6568–6575. doi: 10.4049/jimmunol.1003756
56. Alfredsson J, Wick MJ. Mechanism of fibrosis and stricture formation in Crohn's disease. *Scand J Immunol* (2020) 92:e12990. doi: 10.1111/sji.12990
57. Knight V, Tchongue J, Lourensz D, Tipping P, Sievert W. Protease-activated receptor 2 promotes experimental liver fibrosis in mice and activates human hepatic stellate cells. *Hepatology* (2012) 55:879–887. doi: 10.1002/hep.24784
58. Chrysanthopoulou A, Mitroulis I, Kambas K, Skendros P, Kourtzelis I, Vradelis S, Kolios G, Aslanidis S, Doumas M, Ritis K. Tissue factor-thrombin signaling enhances the fibrotic activity of myofibroblasts in systemic sclerosis through up-regulation of endothelin receptor A. *Arthritis Rheum* (2011) 63:3586–3597. doi: 10.1002/art.30586
59. Arelaki S, Arampatzioglou A, Kambas K, Papagoras C, Miltiades P, Angelidou I, Mitsios A, Kotsianidis I, Skendros P, Sivridis E, et al. Gradient Infiltration of Neutrophil Extracellular Traps in Colon Cancer and Evidence for Their Involvement in Tumour Growth. *PLoS One* (2016) 11:e0154484. doi: 10.1371/journal.pone.0154484
60. Mitroulis I, Kambas K, Chrysanthopoulou A, Skendros P, Apostolidou E, Kourtzelis I, Drosos GI, Boumpas DT, Ritis K. Neutrophil extracellular trap formation is associated with IL-1β and autophagy-related signaling in gout. *PLoS One* (2011) 6:e29318. doi: 10.1371/journal.pone.0029318
61. Larson CL, Shah DH, Dhillon AS, Call DR, Ahn S, Haldorson GJ, Davitt C, Konkel ME. Campylobacter jejuni invade chicken LMH cells inefficiently and stimulate differential expression of the chicken CXCLi1 and CXCLi2 cytokines. *Microbiology (Reading)* (2008) 154:3835–3847. doi: 10.1099/mic.0.2008/021279-0
